# Thyme-synthesized silver nanoparticles mitigate immunosuppression, oxidative damage, and histopathological alterations induced by multidrug-resistant *Enterococcus faecalis* in *Oreochromis niloticus*: in vitro and in vivo assays

**DOI:** 10.1007/s10695-025-01560-5

**Published:** 2025-08-15

**Authors:** Dalia A. Abdel-moneam, Hanan S. Khalefa, Maha M. Rashad, Ghada E. Ali, Yasmine H. Ahmed, Eman Ragab, Osama A. Fouad, Ramadan A. Geioushy, Sahr B. Mahmoud

**Affiliations:** 1 Department of Aquatic Animal Medicine and Management, Faculty of Veterinary Medicine, Cairo University, Giza, 12211 Egypt; 2 Department of Veterinary Hygiene and Management Faculty of Veterinary Medicine, Cairo University, Giza, 12211 Egypt; 3 Department of Biochemistry and Molecular Biology, Faculty of Veterinary Medicine, Cairo University, Giza, 12211 Egypt; 4 Department of Cytology and Histology, Faculty of Veterinary Medicine, Cairo University, Giza, 12211 Egypt; 5 Department of Microbiology, Faculty of Veterinary Medicine, Cairo University, Giza, 12211 Egypt; 6 Nanostructured Materials and Nanotechnology Department, Advanced Materials Institute, Central Metallurgical Research and Development Institute, P.O. Box 87, Helwan, 11421 Cairo Egypt; 7 Department of Hydrobiology, Veterinary Research Institute, National Research Centre, Dokki, Giza, Egypt

**Keywords:** Thyme-AgNPs, *Enterococcus faecalis*, Multidrug-resistant, Nile tilapia, Oxidant/antioxidant, Immune gene expression, Histochemistry

## Abstract

The escalating prevalence of antibiotic resistance poses a significant challenge to controlling aquatic diseases, underscoring the need for innovative antimicrobial approaches. Therefore, the current study aimed to investigate the effects of thyme-synthesized silver nanoparticles (thyme-AgNPs) on modulating the immunosuppressive, oxidative stress, and histopathological changes induced by multidrug-resistant *Enterococcus faecalis* (MDR *E. faecalis*) infection in *Oreochromis niloticus* (Nile tilapia)*.* The antibiotic susceptibility profiling of the isolated *E. faecalis* (PQ084785) revealed resistance to multiple antibiotic classes. The in vitro bactericidal efficacy of thyme-AgNPs was confirmed, with minimum bactericidal concentrations (MBC) and minimum inhibitory concentrations (MIC) of 62.5 µg/mL and 31.5 µg/mL, respectively. In vivo, the 96-h median lethal concentration (LC_50_) of thyme-AgNPs was determined to be 1 mg/L. Meanwhile, eighty *O. niloticus* (30.00 ± 5.00 g) were categorized into four groups in duplicate: group I was the negative control group, group II was intraperitoneally injected with MDR *E. faecalis* (0.2 mL of 0.79 × 10^8^ CFU/mL), group III was exposed to the sublethal dose of thyme-AgNPs in water (0.1 mg/L), and group IV was challenged with the *E. faecalis* and exposed to thyme-AgNPs simultaneously for 15 days. Results outlined that *E. faecalis* induced clinical abnormalities*,* reduced immunoglobulin M levels, elevated oxidant indicators (malondialdehyde), decreased antioxidant capacity (reduced glutathione content, catalase, and superoxide dismutase), and significantly upregulated immune gene expression (tumor necrosis factor-α and interleukin-1β) with marked histopathological and histochemical alterations in gill and liver tissues. Interestingly, the thyme-AgNPs intervention resulted in a noticeable improvement in all measured biomarkers as well as histopathological regeneration. Overall, thyme-AgNPs are a recent antibacterial tool against immune-oxidant/antioxidant and histopathological changes triggered by MDR *E. faecalis* infection.

**Supplementary Information:**

The online version contains supplementary material available at 10.1007/s10695-025-01560-5.

## Introduction

Aquaculture is the fastest-growing food industrial sector, playing a crucial role in mitigating the increasing demand for animal protein by increasing the density of cultivation and production (Mansour et al. 2021). However, fish intensification may cause stress and evoke the outbreak of various diseases (Maulu et al. 2021). The *Oreochromis niloticus* (Nile tilapia) is a significant farmed fish in Egypt, renowned for its delicious flavor and high nutritional value (Sherif and Zommara 2024). Globally, Nile tilapia is now the second most cultivated fish species, with an annual aquaculture production of approximately 5.3 million tons (FAO 2024). Bacterial infections pose a significant challenge to the aquaculture industry, negatively impacting both fish production and overall fish health, resulting in substantial economic losses (Mishra et al. 2018; Khalefa et al. 2021).

Enterococcosis has emerged as one of the devastating and globally significant bacterial fish diseases affecting both marine and freshwater fish, resulting in high mortalities at all stages of culturing (Rahman et al. 2017; Akter et al. 2021). The disease is caused by different groups of bacteria, including *Streptococcus* spp., *Lactococcus* spp., and *Enterococcus* spp., with *Enterococcus faecalis* (*E. faecalis*) currently recognized as the primary causative agent of streptococcosis-like infections in fish in Egyptian waters (Abu-Elala et al. 2020). Moreover, it has been designated as a healthcare-associated pathogen with significant public health implications (Khan et al. 2015) due to its ability to acquire extensive resistance to many commercially available antibiotics, making traditional treatments challenging (Belgacem et al. 2010; Hammerum et al. 2010).

Silver nanoparticles (AgNPs) have been identified as one of the most essential and unique metallic nanoparticles used in biomedical applications, including fisheries, as antibacterial agents (Shaalan et al. 2016; Dahdouh et al. 2020). The bactericidal efficacy of AgNPs was detected against several Gram-negative bacteria, such as *Aeromonas hydrophila*, *Aeromonas veronii*, *Pseudomonas aeruginosa*, *Yersinia ruckeri*, *Vibrio fluvialis*, and *Vibrio harveyi*, as well as Gram-positive *Staphylococcus aureus*, *Streptococcus iniae*, and *Lactococcus garvieae* in different fish species (Soltani et al. 2009; Kanwal et al. 2019; Elgendy et al. 2022). The mechanism of bacterial inhibition by AgNPs begins once they are attached to the bacterial cell surface, where Ag^+^ ions are released and reactive oxygen species (ROS) are generated. This can cause membrane instability, breakdown, and cytoplasmic leakage, leading to damage to the subcellular structure and inactivation of microbial enzymes, proteins, and nucleotides (More et al. 2023). Additionally, AgNPs have been revealed to have the potential to enhance the survival rate, improve physiological status, and reduce both infectious and non-infectious stressors in aquatic animals (Kumar et al. 2019).

AgNPs can be synthesized using physical, chemical, and biological methods (Ijaz et al. 2020). The biological synthesis, known as green or biosynthesis, of NPs has become a more popular and commonly used synthesis approach in nanoscience. Besides being eco-friendly and not generating or utilizing toxic materials, it can be conducted under standard laboratory conditions without incurring significant costs or consuming extensive energy, making it superior to chemical and physical approaches (Tariq et al.^2020^; Nugroho et al. 2022).

Green synthesis utilizes a range of biological organisms, including microbes (bacteria, viruses, fungi, and algae), as well as plants or their extracts. *Thymus vulgaris L.* (thyme) is an aromatic medicinal plant belonging to the family *Lamiaceae* that has potent antimicrobial properties (Dorojan et al. 2015; de Melo et al.^2020^). The major phenolic component of thyme is thymol, which is widely used in aquaculture as a phytogenic essential oil feed supplement that exhibits therapeutic and beneficial effects as an antibacterial, immunostimulant, antioxidant, growth-promoting, and anti-inflammatory agent (Abd El-Hamid et al. 2021; Al-Jahani 2021; Hashem et al. 2022). Several studies have documented that biosynthesized AgNPs can act as an effective antimicrobial agent for controlling a broad range of fish pathogens (Fakharzadeh et al. 2020; Nangare and Patil 2020; Chand et al. 2020; Hussain et al. 2023; Dube 2024; Ramya et al. 2024).

To the best of the researchers’ knowledge, the antimicrobial action of green-synthesized AgNPs using thyme essential oil against multidrug-resistant *Enterococcus faecalis* (MDR *E. faecalis*) in *O. niloticus* has not been investigated yet. Consequently, the objective of this study was to evaluate the in vitro and in vivo bactericidal efficacy of thyme-AgNPs against MDR *E. faecalis*, focusing on the immunological response, oxidative stress parameters, and histopathological and histochemical alterations in challenged fish.

## Materials and methods

### Bacterial strain and culture conditions

Previously, *E. faecalis* was isolated from a tilapia fish mass mortality episode in Sharkia Governorate, Egypt. Infected fish exhibited septicemic symptoms (exophthalmia, red spots, and ascites). Bacteria were isolated on KF streptococcal agar medium (HiMedia, India) from the liver, kidney, and brain of the infected fish. The isolates were subcultured on tryptic soy agar (TSA) (LabM, UK) and maintained in brain heart infusion broth (BHI) (LabM, UK). Preliminary phenotypic identification of the isolates was performed using standard morphological and physiological methods, followed by biochemical analysis with the Rapid HiStrepTM identification testing kit (HiMedia, India) (Devriese et al. 1993).

***16S rRNA*** **gene amplification and sequencing analysis**

The procedure for extracting bacterial genomic DNA was performed as described by Dong et al. (2015). The 16S ribosomal RNA gene was amplified using universal prokaryotic primers F: 5′-AGAGTTTGATCCTGGCTCAG-3′ and R: 5′-GGTTACCTTGTTACGACTT-3′ following Eden et al. (1991). The PCR products were resolved on a 1.5% agarose gel containing 0.5 μg/mL ethidium bromide in 1X Tris–borate-EDTA (TBE) buffer, and then, they were electrophoresed at 100 V. The amplified PCR product was purified using the DNA Clean and Concentrator®-25 (Zymo Research products, USA), and the sequencing analysis was performed using an ABI 3730xl DNA sequencer (Applied Biosystems™) at Sigma Scientific Services Laboratory (Cairo, Egypt). The obtained sequence was blasted against the NCBI database. Based on alignments of *16S rRNA* locus sequences, a phylogenetic tree was constructed using the MEGA 11 software employing the neighbor-joining method (Kumar et al. 2018).

### Antibiotic susceptibility profile

The susceptibility profile of the pathogenic *E. faecalis* isolate to various commercial antibiotics was determined using the disk diffusion method on Mueller–Hinton agar (MHA) (Bauer et al. 1966). The tested antibiotic discs (Oxoid) were chloramphenicol (C, 30 µg), ciprofloxacin (CIP, 5 µg), erythromycin (E, 15 µg), gentamicin (CN, 10 µg), levofloxacin (LEV, 5 µg), streptomycin (S, 10 µg), tetracycline (TE, 30 µg), trimethoprim–sulfamethoxazole (SXT, 25 µg), penicillin (P, 10 µg), clindamycin (DA, 2 µg), and nitrofurantoin (F, 300 μg). Antibiotic disks were aseptically placed on the culture plate and incubated at 37 °C for 24 h. After incubation, the diameter of the zone of inhibition was measured. The results were categorized as sensitive (S), intermediate (I), and resistant (R) according to the Clinical and Laboratory Standards Institute (CLSI 2021), which defines multidrug-resistant (MDR) bacteria as those that are resistant to at least three antimicrobial classes (Magiorakos et al. 2012).

### Synthesis and characterization of green AgNPs

#### Thyme-AgNP synthesis

The green synthesis of AgNPs was conducted by using thyme essential oil (Cap Pharm, Egypt). A solution of silver nitrate (AgNO_3_) was prepared, and the pH of the solution was adjusted to 8 by adding 0.1 M NaOH and heating to 60 °C while stirring continuously. Afterwards, thyme oil was added dropwise until the solution changed from brown to yellow, indicating that the surface plasmon resonance phenomenon had occurred and confirming that AgNPs had been successfully synthesized.

#### Thyme-synthesized AgNPs characterization

To verify the green synthesis of AgNPs, UV/VIS/NIR spectrophotometry analysis was performed in scanning mode using a Jasco-V-770 UV/VIS/NIR spectrophotometer (Japan) in the range of 300–800 nm. Additionally, the size and shape of the green-synthesized NPs were analyzed using high-resolution transmission electron microscopy (HR-TEM) with a JEOL-JEM-1230 microscope (Tokyo, Japan). The TEM samples were prepared by placing a drop of AgNPs suspension on a 400-mesh carbon-coated copper grid and allowing the solvent to evaporate in the air at room temperature. A micro-focused monochromator X-ray photoelectron spectroscopy (XPS), Thermo Scientific™ K-Alpha™ (up to 4 keV), was used to identify the valence state and elemental composition.

### In vitro evaluation of the antibacterial activity of thyme-AgNPs

#### Agar diffusion assay

The effect of thyme-AgNPs on *E. faecalis* was assessed using the agar well diffusion method (Hamida et al. 2020a). Growth inhibition tests were conducted using MHA (Biomerieux, France) to evaluate the effects of enriched culture medium on growth. Freshly cultured 24-h *E. faecalis* was streaked on an MHA plate. This plate was perforated to form wells of 5 mm diameter, which were aseptically filled with 100 μL of different concentrations of thyme-AgNPs (15.5, 31.25, 62.5, 125, 250, 500, and 1000 μg/mL) and distilled water (as a negative control). Following incubation at 37 °C for 24 h, inhibition zones were measured under reflected light in millimeters (mm) using a transparent ruler.

#### Minimum inhibitory concentrations and minimum bactericidal concentrations

As per the CLSI document (CLSI 2012), the MIC and MBC were determined in Mueller–Hinton (MH) broth medium. Tubes of serially diluted thyme-AgNPs (1000 to 15.5 μg/mL) were equally inoculated with 100 μL of a freshly prepared bacterial suspension (10^6^ CFU/mL), followed by overnight incubation. Following the observation of turbidity and the presence or absence of microorganism growth in the tubes, the MIC was determined. For the evaluation of reproducibility, some tests were conducted in triplicate. The MBC was determined by subculturing 50 μL from each test tube, which displayed no apparent growth (clear) on MHA plates, and maintaining the temperature at 37 °C for 24 h. The plates were examined for macroscopic bacterial growth to determine whether the growth was present or not. MBC endpoints are achieved when 99.9% of the bacterial population is killed at the lowest concentration of a nanoparticle agent (Parvekar et al. 2020). Based on the MBC/MIC ratio, silver derivatives were classified as bacteriostatic or bactericidal depending on the score (Ayala-Núñez et al. 2009).

### In vivo antibacterial activity of thyme-AgNPs

#### Experimental fish

One hundred and thirty *O. niloticus* fish with an average weight of 30.00 ± 5.00 g were obtained from a private fish farm in Al-Sharqia Governorate. Fish were transported in fiberglass tanks equipped with oxygen aerators to the laboratory, where they were acclimated for 2 weeks in plastic containers (66 cm × 47 cm × 42 cm) before the start of the experimental study. Fish were maintained in a laboratory environment under identical conditions of water temperature (27 ± 1.09 °C), pH (6.9 ± 0.5), dissolved oxygen (6.5 ± 0.60 mg/L), and ammonia (0.030 ± 0.02 mg/L) with a controlled photoperiod (12:12 h light/dark).

#### In vivo LC_50_ evaluation of thyme-AgNPs

After acclimation, fifty *O. niloticus* fish were divided into five groups (10 fish in each) and exposed to different concentrations of prepared thyme-AgNPs (0, 1, 1.3, 1.5, and 1.8 mg/L) for the detection of the 96-h median lethal concentration LC_50_ under laboratory conditions (Younas et al. 2022). Mortalities were recorded at 24, 48, 72, and 96 h; dead fish were promptly removed to prevent any changes in the water parameters. The LC_50_ was calculated with a 95% confidence limit using the probit analysis method (Finney 1971). For sublethal studies, one-tenth of the 96-h LC_50_ concentration was determined (0.1 mg/L).

#### Experimental design

**Fig. 1:**
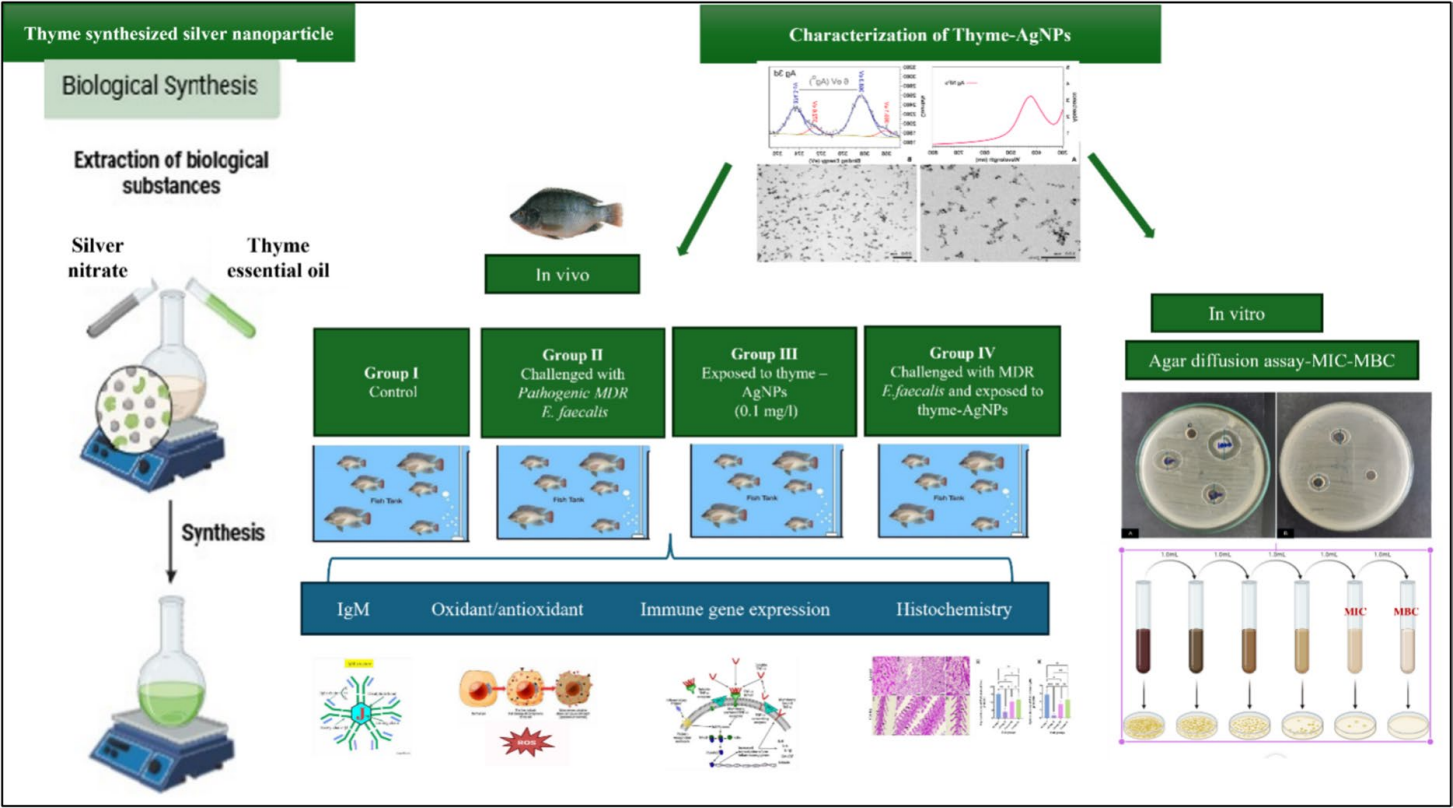
Experimental scheme

Eighty *Nilotica* fish were categorized into four groups in duplicate (*n* = 10). Group I was a negative control group; group II was intraperitoneally injected with 0.2 mL of a pathogenic MDR *E. faecalis* strain at a dose of 0.79 × 10^8 CFU^/mL (Rizkiantino et al. 2021); group III was exposed to the sublethal dose of thyme-synthesized AgNPs (thyme-AgNPs) in water (0.1 mg/L); and group IV was injected with pathogenic *E. faecalis* bacteria and concurrently exposed to thyme-AgNPs as illustrated in Fig. 1. During the 15-day experimental period, the fish were fed twice daily (at 9 a.m. and 3 p.m.) on a basal diet containing 30% protein. Water was exchanged every 48 h, accompanied by the addition of a new dose of thyme-AgNPs, to reduce the buildup of metabolic waste and maintain constant NP concentrations.

### Immunological assay

The level of immunoglobulin M (IgM) was measured in fish serum at the end of the experimental study. Five fish per group were randomly harvested and anaesthetized with 50 mg/L MS222 (Sherif et al. 2023). Blood samples were collected without an anticoagulant using a 2.5-mL syringe from the caudal vein, which was then centrifuged at 3000 rpm for 15 min. Next, serum samples from all groups were separated and tested for IgM levels using the ELISA technique, as per the manufacturer’s instructions (Biocheck Inc., Foster City, CA, USA). Optical density was measured spectrophotometrically at 490 nm using a SpectraMax 190 spectrophotometer (Molecular Devices, Sunnyvale, CA, USA). The concentration of IgM in the samples was then determined by comparing the O.D. of the samples to that of the control, and the IgM concentration was expressed as mg/dl.

### Oxidative stress biomarkers

Gill and liver tissues were homogenized in ice-cold 0.1 M phosphate buffer saline (pH = 7.4) using a Teflon tissue homogenizer. The crude tissue homogenate was centrifuged at 15,000 r/min for 15 min at 4 °C and used for measurement of reduced glutathione content (GSH) (nmol/gm tissue) (Cat. numbers: GR 2511) using the reduced glutathione colorimetric method and malondialdehyde (MDA) (nmol/gm tissue) (Cat. numbers: MD 2529) using the thiobarbituric acid method spectroscopically at 532 nm, using the reagent kits of Bio-diagnostic Co., Giza, Egypt, following the instructions provided (Abdel-Radi et al. 2022).

### Antioxidant-related genes and immune-related genes transcription levels

The relative mRNA abundance of gills, liver catalase (*CAT*), superoxide dismutase (*SOD*), tumor necrosis factor-α (*TNF-α*), and interleukin-1β (*IL-1β*) was determined by PCR amplification of cDNA samples, using the housekeeping gene glyceraldehyde-3-phosphate dehydrogenase (*GAPDH*) (Ahmed et al. 2025; Abd El Megeed et al. 2025). Approximately 50 mg of gill and liver tissues were used for total RNA extraction using a total RNA extraction kit. The concentration and purity of RNA were confirmed, and then, RT-PCR was performed using M-MuLV Reverse Transcriptase (NEB #M0253). A fluorescence-based real-time detection method using the fluorescent SYBR green dye (Thermo Scientific, Cat. No. K0221) was employed for the quantitative assessment of DNA amplification for each gene. The primer sequences used to amplify the selected genes are presented in Table 1. Changes in the product concentrations were assessed by measuring the fluorescence level during the elongation phase of PCR. The real-time PCR conditions were performed as described by Abdel-moneam et al. (2025a). In each experiment, negative controls were included that were free of the template. Each qRT-PCR was performed with three biological replicates, and each biological replicate was assessed three times to ensure the robustness of our results (Abdelkhalek et al. 2024). The relative transcription levels of each gene were calculated using the comparative 2^−ΔΔCT^ method (Livak and Schmittgen 2001).

**Table 1:** The primer sequences used to amplify the study’s genes

Gene*	Acc. no	Forward	Reverse
*GAPDH*	NM_001279552.1	GCTGTACATGCACTCCAAGG	ACTCAAACACACTGCTGCTG
*CAT*	JF801726.1	AGAACTTGGCCGGGTTTCTA	CGGCTGTAAACGTGCAAAGT
*SOD*	JF801727.1	CCCTACGTCAGTGCAGAGAT	GCCGCCTCCATTAAACTTGA
*TNF-α*	NM_001279533.1	GCCTCACAATTCTCAGCCAC	AAACACGCCAAAGAAGGTCC
*IL-1β*	KF747686.1	CACAAGGATGACGACAAGCC	TCTCCTGACACACTTCCACC

*GAPDH, glyceraldehyde-3-phosphate dehydrogenase; *CAT*, catalase; *SOD*, superoxide dismutase; *TNF-α*, tumor necrosis factor-α; *IL-1β*, interleukin-1β

### Histopathological investigations

#### Light microscopic examination

Gill and liver tissues were removed from the fish in all experimental groups and fixed in 10% NBF. The tissues were dehydrated in ascending grades of alcohol, cleared in xylene, and embedded in paraffin. Four-micrometer thick tissue sections were obtained using a rotary microtome. The sections were then stained with hematoxylin and eosin (H&E) for histopathological investigations (Bancroft and Gamble 2013).

#### Histochemical examination

Paraffin sections of the gills and liver were stained with a periodic acid-Schiff (PAS) stain to demonstrate neutral mucopolysaccharides (Bancroft and Gamble 2013). Periodic acid-Schiff sections were analyzed using a digital Leica Quin 500Â image analysis system (Leica Microsystems, Switzerland) housed at the Faculty of Dentistry, Cairo University. Histochemical staining is presented as optical density in a standard measuring frame, averaged over ten independent fields from different slides in each group, at × 400 magnification. All areas with positive histochemical results were chosen for evaluation.

### Statistical analysis

Data were analyzed and expressed as mean ± SE, using one-way analysis of variance (ANOVA) to determine the significance of the mean differences between groups, followed by Tukey’s post hoc test. A *p-*value < 0.05 was considered statistically significant, and the data were graphed as bar charts using GraphPad Prism 10 (GraphPad Software, USA).

## Results

### Bacterial identification

Morphological studies revealed that the *E. faecalis* isolate produced small- to medium-sized, circular, smooth, and raised colonies on KF streptococcal agar plates. All of them formed dark red-colored colonies on KF streptococcal agar medium, but formed creamy, transparent-colored colonies when grown on TSA. All isolates were Gram-positive cocci, non-motile, catalase- and oxidase-negative, glucose-fermentative, esculin-hydrolyzing, and Voges-Proskauer-positive. The biochemical identification results of the Rapid HiStrep™ Tests are presented in Table S1.

### Phylogenetic analysis

**Fig. 2:**
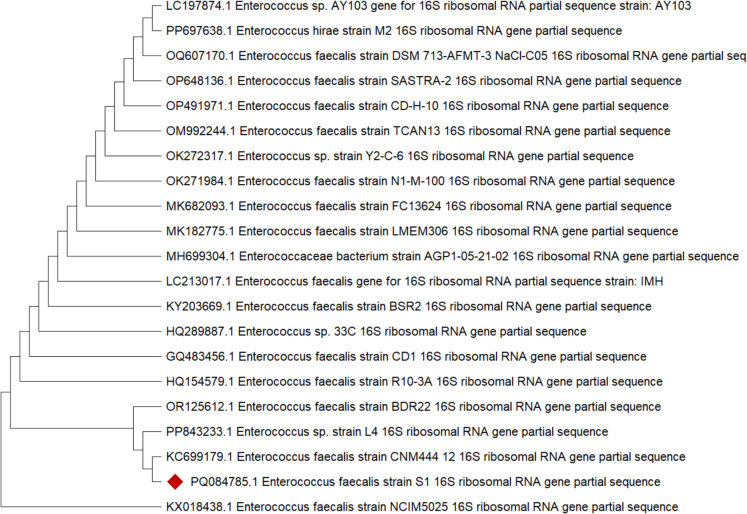
The constructed phylogenetic tree based on the partial *16S rRNA* gene sequence of *E. faecalis* using neighbors-joining method

Molecular identification using the universal *16S rRNA* gene confirms the isolation of *E. faecalis*. The sequence of amplified DNA fragments was registered in GenBank under accession number PQ084785. The sequence alignment and phylogenetic analysis revealed that the sequenced isolate was placed in the same clade and shared the same ancestors in the genus *Enterococcus* with a high identity percentage (99.57%) (Fig. 2).

### Antibiotic susceptibility profile and multidrug-resistance detection

*E. faecalis* isolate demonstrated resistance to multiple antibiotics such as erythromycin, gentamicin, streptomycin, tetracycline, and clindamycin. The isolate was susceptible to chloramphenicol, ciprofloxacin, levofloxacin, trimethoprim–sulfamethoxazole, and penicillin while displaying intermediate sensitivity to chloramphenicol and nitrofurantoin (Figure S1).

### Characterization of thyme-AgNPs

**Fig. 3:**
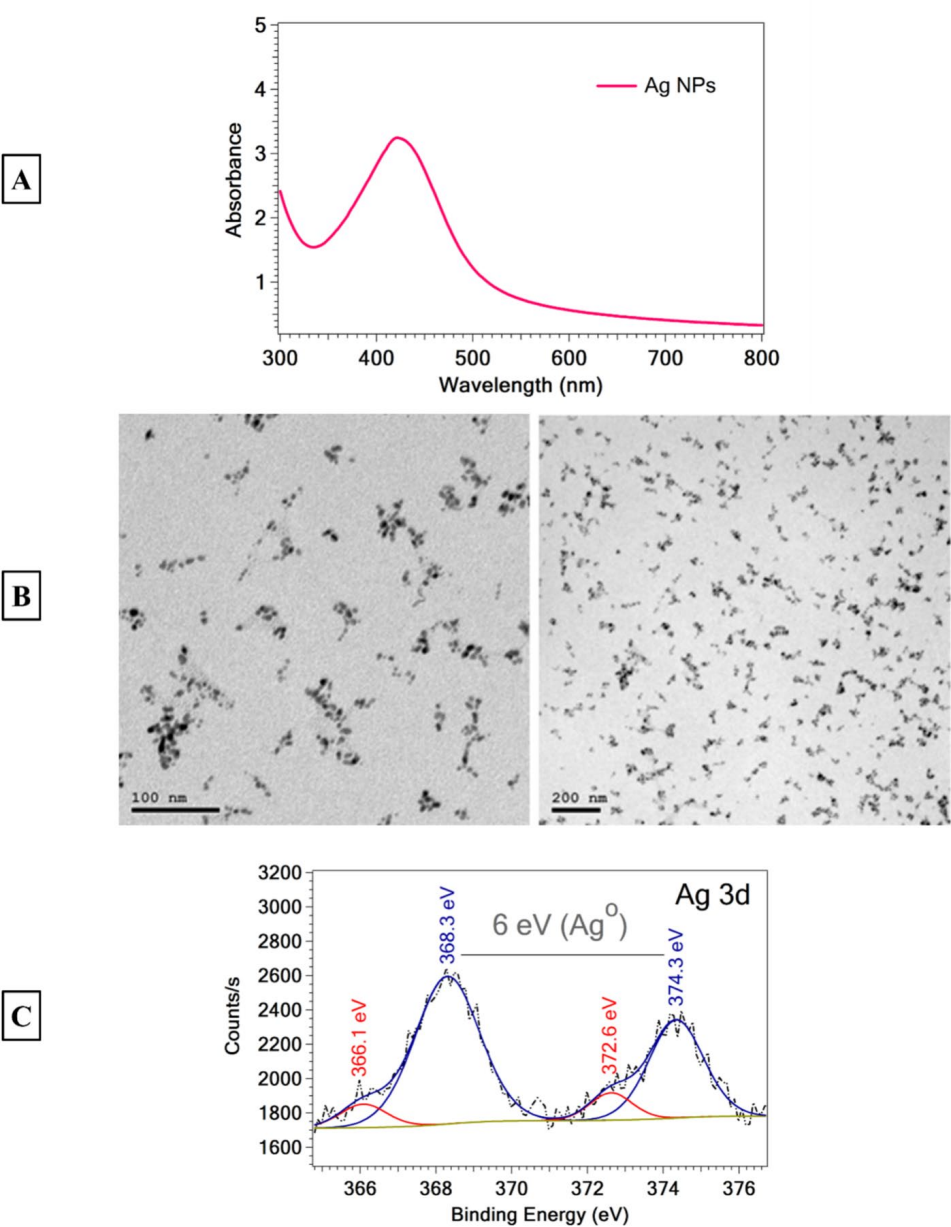
**A)** UV–vis absorption spectra, **B)** TEM images of AgNPs, and **C)** high-resolution XPS spectra of Ag 3d of AgNPs sample synthesized with thyme oil

Figure 3 illustrates the optical properties of AgNPs synthesized through a green method. UV–vis spectroscopy is a widely used technique for characterizing these nanoparticles. Silver particles with an average diameter ranging from 35 to 80 nm exhibit a broad absorption peak around 440 nm (Budhiraja et al. 2013), as depicted in Fig. 3A. The surface morphology of the thyme-synthesized AgNPs was studied by TEM, as illustrated in Fig. 3B. AgNPs were formed in spherical shapes with a particle size of ~ 10 nm. Moreover, the synthesized AgNPs are surrounded by an organic layer, specifically thyme oil. This layer plays a crucial role in stabilizing the particles and effectively preventing agglomeration.

The successful formation of AgNPs in the metallic form was confirmed by XPS analysis, as illustrated in Fig. 3C. The deconvolution of the Ag 3d revealed two peaks positioned at binding energy values of 374.3 and 368.3 eV. These two peaks correspond to Ag^0^ 3d_3/2_ and Ag^0^ 3d_5/2_, respectively. Additionally, the energy difference between the two peaks is 6 eV, indicating the oxidation state of metallic silver (Ag^0^) (Sharma et al. 2018). Moreover, there are two peaks at binding energy values of 372.6 and 366.1 eV, which are ascribed to traces of Ag^+^. The data confirmed that Ag^+^ was reduced to Ag^0^ by protons donated from the functional groups in thyme oil (de Melo et al. 2020).

### In vitro antibacterial activity of thyme-AgNPs

#### Growth inhibition by agar diffusion assay

All inhibition zones produced were plotted according to their size, as illustrated in Table 2. The inhibition zones varied according to concentration, with larger zones at concentrations of 1000, 500, 250, 125, and 62 μg/mL, corresponding to zones of 22, 20, 18, 16, and 14 mm, respectively. Figure S2 depicts that the diameter of zones increases when the concentration of NPs increases, where clear inhibition zones were recorded in the concentrations (1000, 500, 250, and 125 μg/mL); no zones were recorded at the minimal concentrations (< 62.5 μg/mL).

**Table 2:** Diameter of inhibition zone (mm) of thyme-AgNPs against pathogenic MDR *E. faecalis*, MIC, and MBC

Concentration (μg/mL)	Zone of inhibition (mean + SE)	MIC (µg/mL)	MBC (µg/mL)	MBC/MIC ratio
1000	22.67 ± 0.088^a^	31.2	62.5	2
500	20.33 ± 0.086^ab^			
250	19.13 ± 0.058^b^			
125	16 ± 0.054^c^			
62.5	14 ± 0.034^c^			
31	0.00 ± 0.00^d^			
15.5	0.00 ± 0.00^d^			
*p* value	0.001			

Values are presented as mean ± SE. Groups in the same columns having different letters are significantly different from each other at *p* < 0.05. Groups with similar letters are not significantly different

#### Detection of MIC and MBC

The MIC is the minimum concentration that inhibits bacterial growth; in serially diluted tubes, it was recorded at a concentration of 31.2 µg/mL. The MBC, or minimal concentration that stops bacterial growth, was recorded at a concentration of 62.5 µg/mL in count plates. The MBC/MIC ratio was used to define the mode of activity of silver derivatives. According to the current results, the compound was considered bactericidal at a concentration of 2 (Figure S3).

### In vivo challenge

#### Detection of proper LC_50_ of thyme-AgNPs

No mortalities were observed in the acclimated fish before thyme-AgNPs exposure. Mortalities were recorded as 50%, 70%, 85%, and 100% after thyme-AgNPs exposure doses of 1, 1.3, 1.5, and 1.8 mg/L, respectively. The LC_50_ value of thyme-AgNPs was 1 mg/L. For sublethal studies, the one-tenth value of the 96-h LC_50_ concentration for thyme-AgNPs was calculated (0.1 mg/L). The sublethal dose revealed no mortality during the study period.

#### Effect of *E. faecalis* and/or thyme-AgNPs on clinical signs and mortality of *O. niloticus*

Fish in group II, which were challenged with *E. faecalis*, revealed various degrees of abnormal swimming movements, skin discoloration, and poor reflexes. Furthermore, signs of septicemia, such as hemorrhagic patches on the abdomen and some ulcerations, were observed**.** The severity of these symptoms was alleviated in group IV, where fish were simultaneously challenged with *E. faecalis* and exposed to thyme-AgNPs*.* However, no abnormal behavior was observed in fish in group III after exposure to a sublethal dose of thyme-AgNPs. The highest mortality rate was observed in group II (100%). In contrast, exposure to thyme-AgNPs in group IV resulted in only 20% fish mortality, while the control and group III did not experience any fish mortality.

#### Effect of *E. faecalis* and/or thyme-AgNPs on the immunological function of *O. niloticus*

The levels of serum IgM were significantly decreased in *E. faecalis-*challenged fish in group II (2.33 ± 0.06) compared to the negative control group (group I) (3.15 ± 0.04) (*p* < 0.05). On the contrary, co-exposure to thyme-AgNPs in group IV resulted in a significant increase in IgM levels to 4.66 ± 0.05 compared to the control and the *E. faecalis*-challenged group (*p* < 0.05). IgM levels in group III, which received thyme-AgNPs only, revealed the highest IgM values with significant upregulation (5.84 ± 0.03) compared to other experimental groups, as illustrated in Table 3.

**Table 3:** The effect of *E. faecalis* and/or thyme-AgNPs on the IgM serum levels and oxidative stress biomarkers

Fish groups	IgM level (mg/dl)	Oxidative stress biomarkers			
		GSH content (nmol/gm tissue)		MDA level (nmol/gm tissue)	
		Gills	Liver	Gills	Liver
Group I	3.15 ± 0.04^c^	6.06 ± 0.44^a^	6.49 ± 0.46^a^	37.80 ± 1.06^c^	48.17 ± 0.49^c^
Group II	2.33 ± 0.06^d^	1.87 ± 0.09^c^	2.30 ± 0.11^c^	67.43 ± 0.86^a^	78.80 ± 4.20^a^
Group III	5.84 ± 0.03^a^	5.57 ± 0.35^a^	6.52 ± 0.51^a^	37.63 ± 0.90^c^	47.63 ± 1.23^c^
Group IV	4.66 ± 0.05^b^	4.10 ± 0.06^b^	4.74 ± 0.18^b^	42.60 ± 1.19^b^	60.33 ± 1.12^b^
*p* value	< 0.05	< 0.05	< 0.05	< 0.05	< 0.05

Values are presented as mean ± SE (*n* = 5 fish/group). Groups in the same columns having different letters are significantly different from each other at *p* < 0.05. Groups with similar letters are not significantly different. Group I: control; group II: fish group challenged with *E. faecalis*; group III: thyme-AgNPs exposed group; group IV: fish group challenged with *E. faecalis* and exposed to thyme-AgNPs

### Effect of *E. faecalis* and/or thyme-AgNPs on the oxidative stress biomarkers

Oxidative stress biomarkers in gill and liver tissues were assessed by determining GSH and MDA levels. *E. faecalis* induced oxidative stress in the gills and liver as indicated by a significant decrease in GSH (1.87 ± 0.09, 2.30 ± 0.11 nmol/gm tissue) and an increase in MDA (67.43 ± 0.86, 78.80 ± 4.20 nmol/gm tissue) in comparison to the negative control group (*p* < 0.05). As illustrated in Table 3, thyme-AgNPs co-exposure in group IV ameliorated the *E. faecalis*-induced oxidative stress by the significant increase in GSH (4.10 ± 0.06, 4.74 ± 0.18 nmol/gm tissue) and decrease in MDA levels (42.60 ± 1.19, 60.33 ± 1.12 nmol/gm tissue) in comparison with the *E. faecalis-*challenged group (*p* < 0.05). The GSH content and MDA levels of the thyme-AgNPs-exposed group (group III) were significantly different from those of groups II and IV but not substantially different from those of the control group.

### Effect of *E. faecalis* and/or thyme-AgNPs on the expression of antioxidant-related genes

**Fig. 4:**
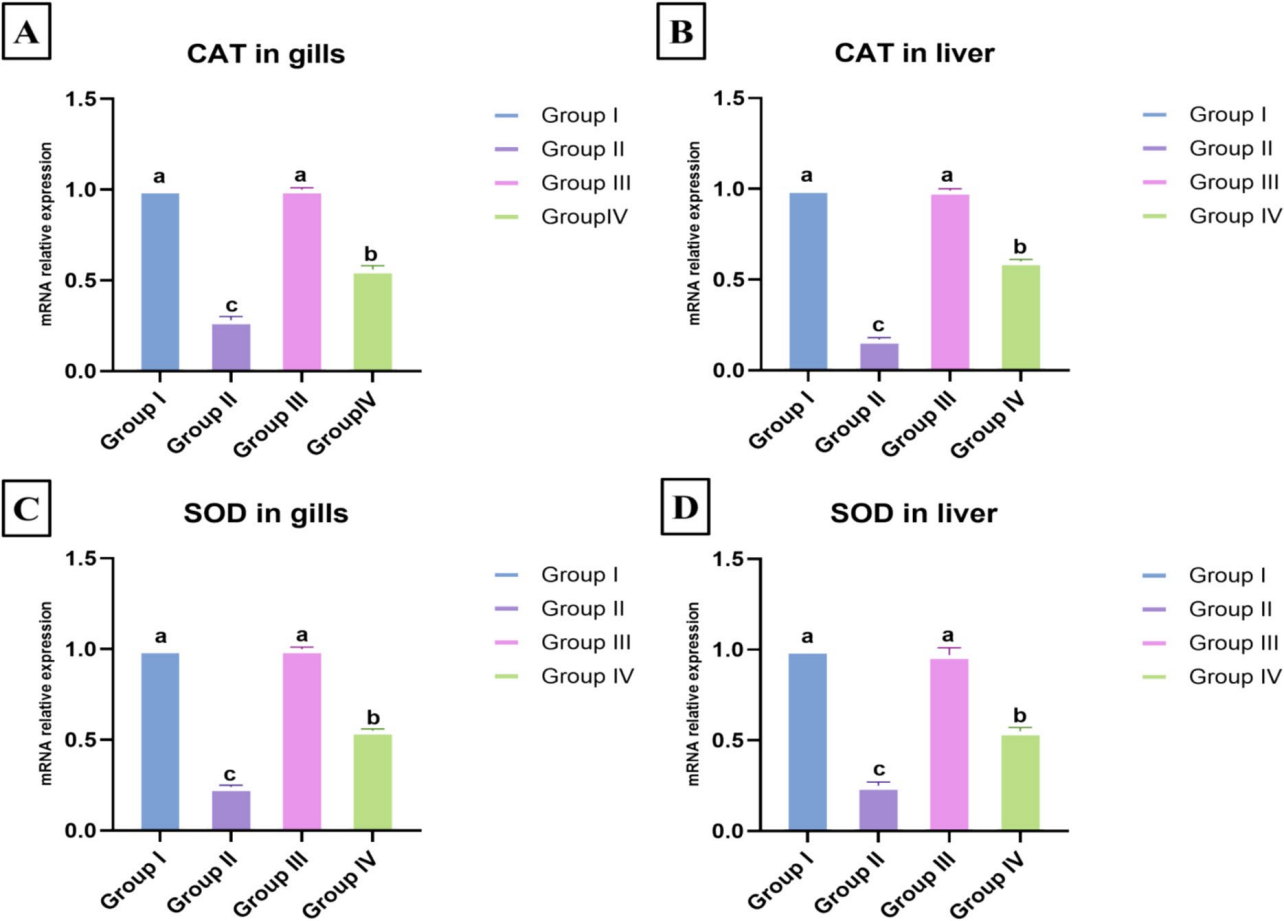
The effect of *E. faecalis* and/or thyme-AgNPs on the mRNA relative expression of antioxidant related genes, *CAT* gene (**A**, **B**) and *SOD* gene (**C**, **D**) in the gills and liver of *O. niloticus*. Values are presented as mean ± SE (*n* = 5 fish/group). Groups having different letters are significantly different from each other at *p* < 0.05. Groups with similar letters are not significantly different. Group I: control; group II: fish group challenged with *E. faecalis*; group III: thyme-AgNPs exposed group; group IV: fish group challenged with *E. faecalis* and exposed to thyme-AgNPs

According to the data obtained in Fig. 4A–B, the expression of the catalase (*CAT)* gene in the gills and liver of *E. faecalis-*challenged fish in group II was significantly downregulated to 0.28- and 0.17-fold, respectively, compared to the control negative group (*p* < 0.05). Thyme-AgNPs exposure in group IV resulted in a significant upregulation of *CAT* expression to 0.56 and 0.60, compared to the *E. faecalis*-challenged group (*p* < 0.05). Similarly, superoxide dismutase (*SOD*) gene expression in the gills (0.24-fold) and liver (0.25-fold) was significantly downregulated upon *E. faecalis* challenge compared to the control group (*p* < 0.05). The antioxidant effect of thyme-AgNPs in group IV was indicated by a significant upregulation of *SOD* gene expression to 0.55-fold in the gills and liver compared to the *E. faecalis* challenged group (*p* < 0.05) (Fig. 4C–D). The *CAT* and *SOD* gene expression values in the gills and liver of thyme-AgNPs (group III) were significantly different from those of groups II and IV, but with values nearly identical to those of the control group (Fig. 4A–D).

### Effect of *E. faecalis* and/or thyme-AgNPs on the immune-related gene transcription levels

**Fig. 5:**
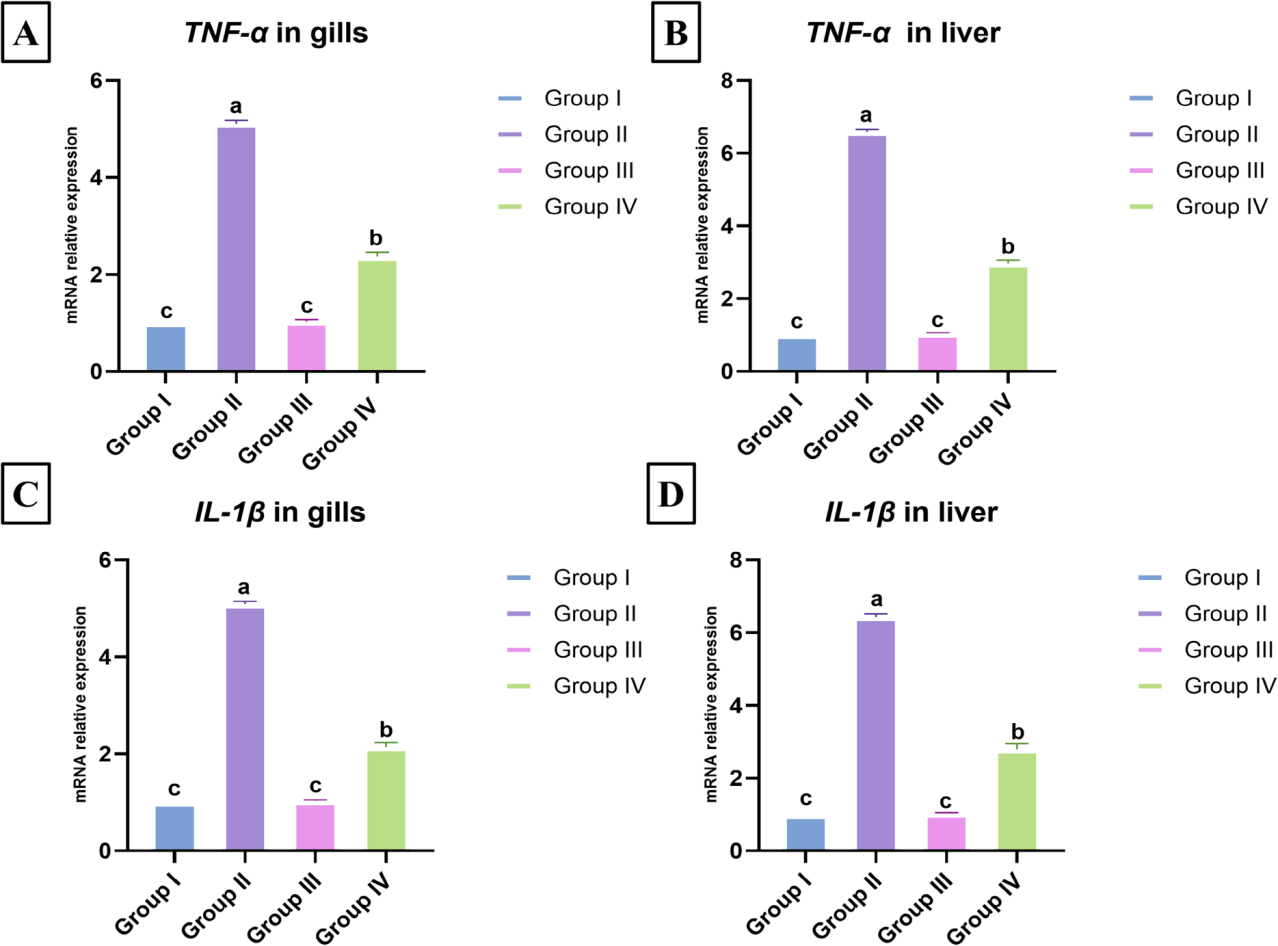
The effect of *E. faecalis* and/or thyme-AgNPs on the mRNA relative expression of immune related *TNF-α* gene (**A**, **B**) and *IL-1β* gene (**C**, **D**) in the gills and liver of *O. niloticus*. Values are presented as mean ± SE (*n* = 5 fish/group). Groups having different letters are significantly different from each other at *p* < 0.05. Groups with similar letters are not significantly different. Group I: control; group II: fish group challenged with *E.faecalis*; group III: thyme-AgNPs exposed group; group IV: fish group challenged with *E.faecalis* and exposed to thyme-AgNPs

In the gill and liver tissues, the expression of the *TNF-α* gene demonstrated a significant upregulation of 5.11- and 6.60-fold, respectively, in the *E. faecalis*-challenged group (group II) compared to the negative control group (*p* < 0.05). In contrast, co-exposure with thyme-AgNPs in group IV induced significant downregulation in the gills and liver gene expression to 2.37- and 2.97-fold, respectively, compared to the *E. faecalis* challenged group (*p* < 0.05), as depicted in Fig. 5A–B. Similarly, the gill and liver *IL-1β* gene expression revealed a significant upregulation of 5.09- and 6.43-fold, respectively, in the *E. faecalis*-challenged group compared to the negative control group (*p* < 0.05). Conversely, thyme-AgNPs co-exposure in group IV significantly downregulated the expression level of the *IL-1β* gene in gills and liver to 2.14- and 2.80-fold, respectively, compared to the *E. faecalis*-challenged group (*p* < 0.05), as displayed in Fig. 5C–D. The *TNF-α* and *IL-1β* gene transcription levels in the gills and liver of thyme-AgNPs (group III) revealed significant differences compared to those of groups II and IV. Still, they were not significantly different from those of the control group (Fig. 5A–D).

### Histopathological investigations

#### Light microscopic observations

**Fig. 6:**
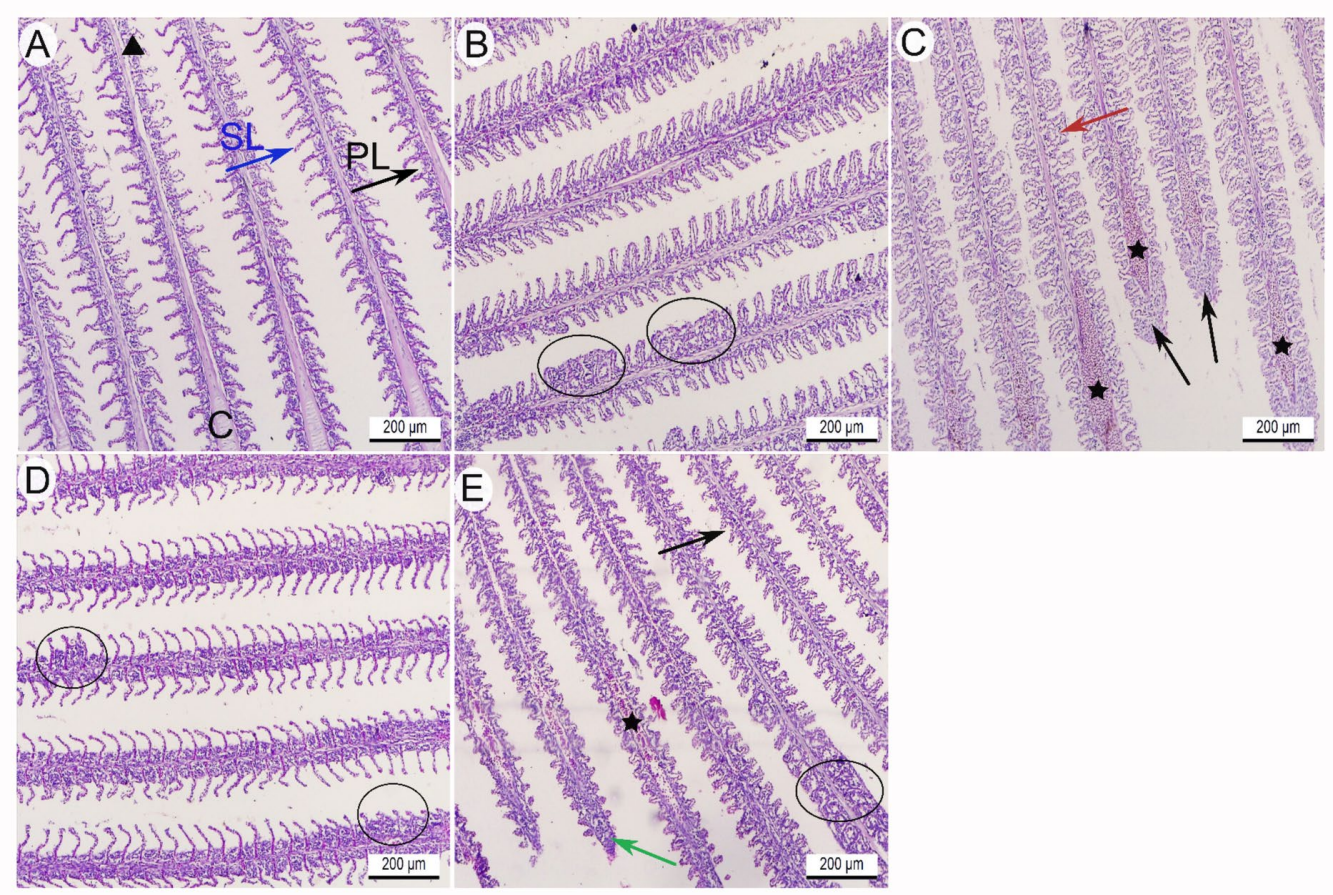
**A**–**E** Gill sections of *O. niloticus*. H&E stain. × 100. **A** Control fish (group I) had a normal histoarchitecture of the gills primary (PL) and secondary lamellae (SL). The primary lamella has a central cartilaginous core (C) and a central venous sinus (black arrowhead). Fish challenged with *E. faecalis* (group II) had **B** fusion of secondary lamellae (black circle) and **C** showed epithelial lifting (red arrow) of both primary and secondary lamellae, dilated, and congested venous sinuses (black star) with epithelial hyperplasia (black arrow) at the tip of the primary lamellae. **D** Fish exposed to thyme-AgNPs in group III showed a few secondary lamellae fusions (black circles) compared to the control fish. **E** *E. faecalis* challenged fish and exposed to thyme-AgNPs in group IV showed marked histoarchitectural recovery in the form of nearly normally arranged primary and secondary lamellae (black arrow), diminished epithelial hyperplasia (green arrow), less dilated and congested venous sinus (black star), and few fused secondary lamellae (black circle)

H&E-stained gill sections of the control group had normal histoarchitecture of the primary and secondary lamellae. Each primary lamella had a central cartilaginous core and central venous sinus (Fig. 6A). The primary lamella is covered by stratified squamous cells, namely pavement cells (PVC), acidophilic chloride cells, and mucous cells. However, gill tissue obtained from *O. niloticus* challenged with *E. faecalis* in group II revealed various structural alterations compared to the control group, such as fusion of secondary lamellae (Fig. 6B), epithelial lifting of both primary and secondary lamellae, and dilated and congested venous sinuses with epithelial hyperplasia at the tip of the primary lamellae (Fig. 6C). Furthermore, gill sections of fish exposed to thyme-AgNPs in group III revealed few secondary lamellae fusions compared to the control group **(**Fig. 6D**)**. Meanwhile, the gill sections of *O. niloticus* that were challenged with *E. faecalis* and exposed to thyme-AgNPs in group IV displayed marked histoarchitecture recovery compared to the bacteria-infected group, and characterized by typically arranged primary and secondary lamellae, diminished epithelial hyperplasia, less dilated and congested venous sinuses, and fewer fused secondary lamellae (Fig. 6E).

**Fig. 7:**
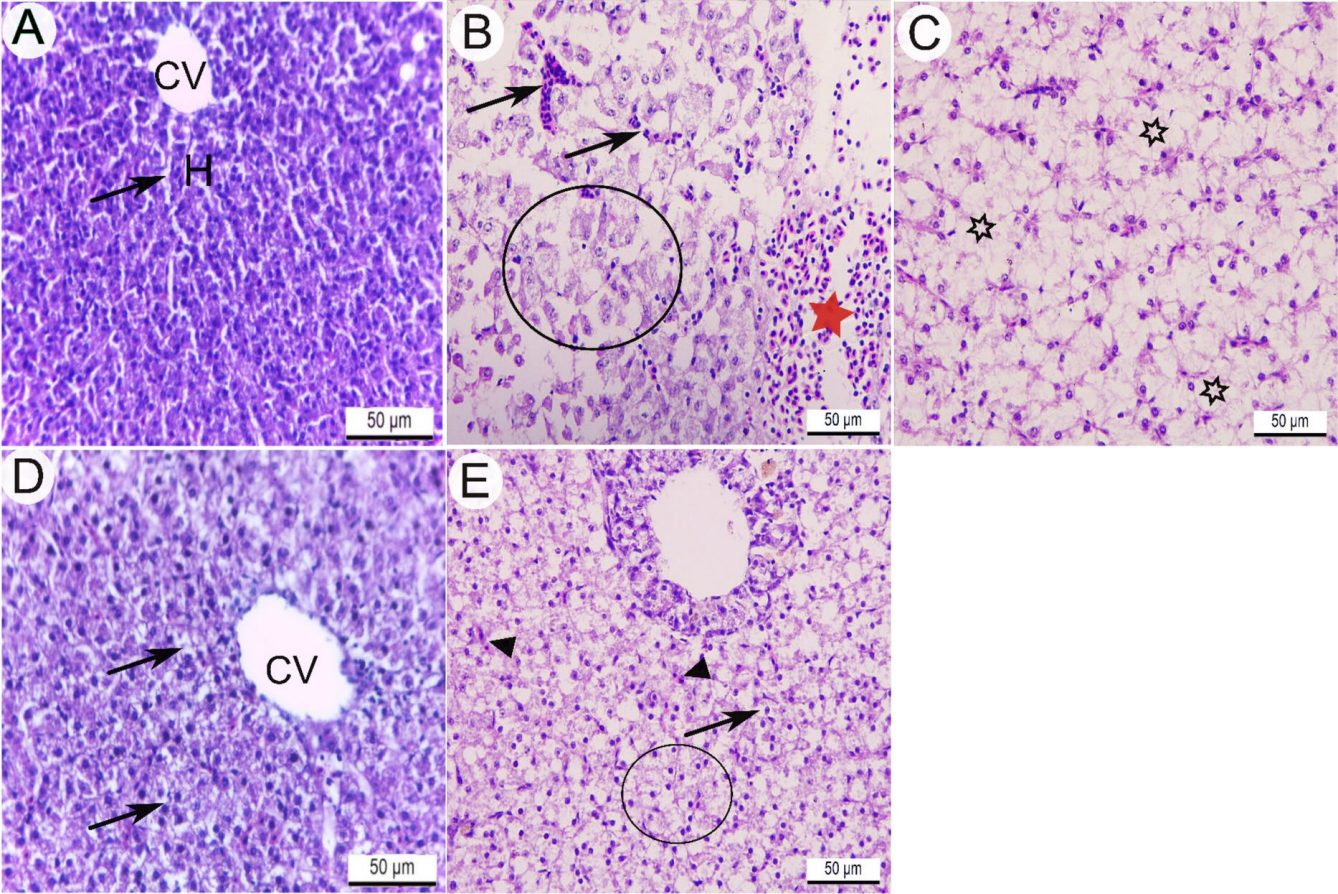
**A**–**E** Liver sections from *O. niloticus*. H&E stain. × 400. **A** Control fish (group I) had normal central vein (CV) and hepatic cords (H) with sinusoids (black arrow) in between. Fish challenged with *E. faecalis* in group II had **B** dilated and congested hepatic sinusoids (black arrows), hepatic cord disintegration (black circle), and hemorrhage (red star) and **C** severe cytoplasmic vacuolar degeneration (black stars). **D** Fish exposed to thyme-AgNPs in group III showed vacuolar degeneration (black arrows) compared with the control group. **E** Fish challenged with *E. faecalis* and exposed to thyme-AgNPs in group IV displayed improvement in hepatic parenchyma structure like narrower and less congested blood sinusoids (black arrowheads) and restoration of hepatic cord arrangement (black circle), except for less cytoplasmic vacuolar degeneration (black arrow)

The liver tissue of the *O. niloticus* control group revealed the standard histological structure of hepatic parenchyma, consisting of the central vein with radiating hepatic cords and hepatic sinusoids in between. Each hepatic cord consisted of polygonal hepatocytes with large vesicular central nuclei (Fig. 7A). In contrast, the hepatic tissue of *E. faecalis*-challenged fish in group II exhibited several histopathological changes in the hepatic parenchyma compared to the control, including dilated and congested hepatic sinusoids, the disintegration of the hepatic cords, and hemorrhage (Fig. 7B). Additionally, hepatocytes exhibited severe cytoplasmic vacuolar degeneration, causing the nuclei to be peripherally situated, resulting in a signet ring appearance (Fig. 7C). The hepatic tissue of fish exposed to thyme-AgNPs in group III displayed hepatocytes with vacuolar degeneration compared to the control (Fig. 7D). In contrast, liver sections of *O. niloticus* that were challenged with *E. faecalis* and exposed to thyme-AgNPs in group IV revealed an improvement in hepatic parenchyma structure compared to group II, and characterized by narrower and less congested blood sinusoids and restoration of hepatic cord arrangement, except for the presence of vacuolar degeneration, albeit to a lesser extent (Fig. 7E).

#### Histochemical investigations

**Fig. 8:**
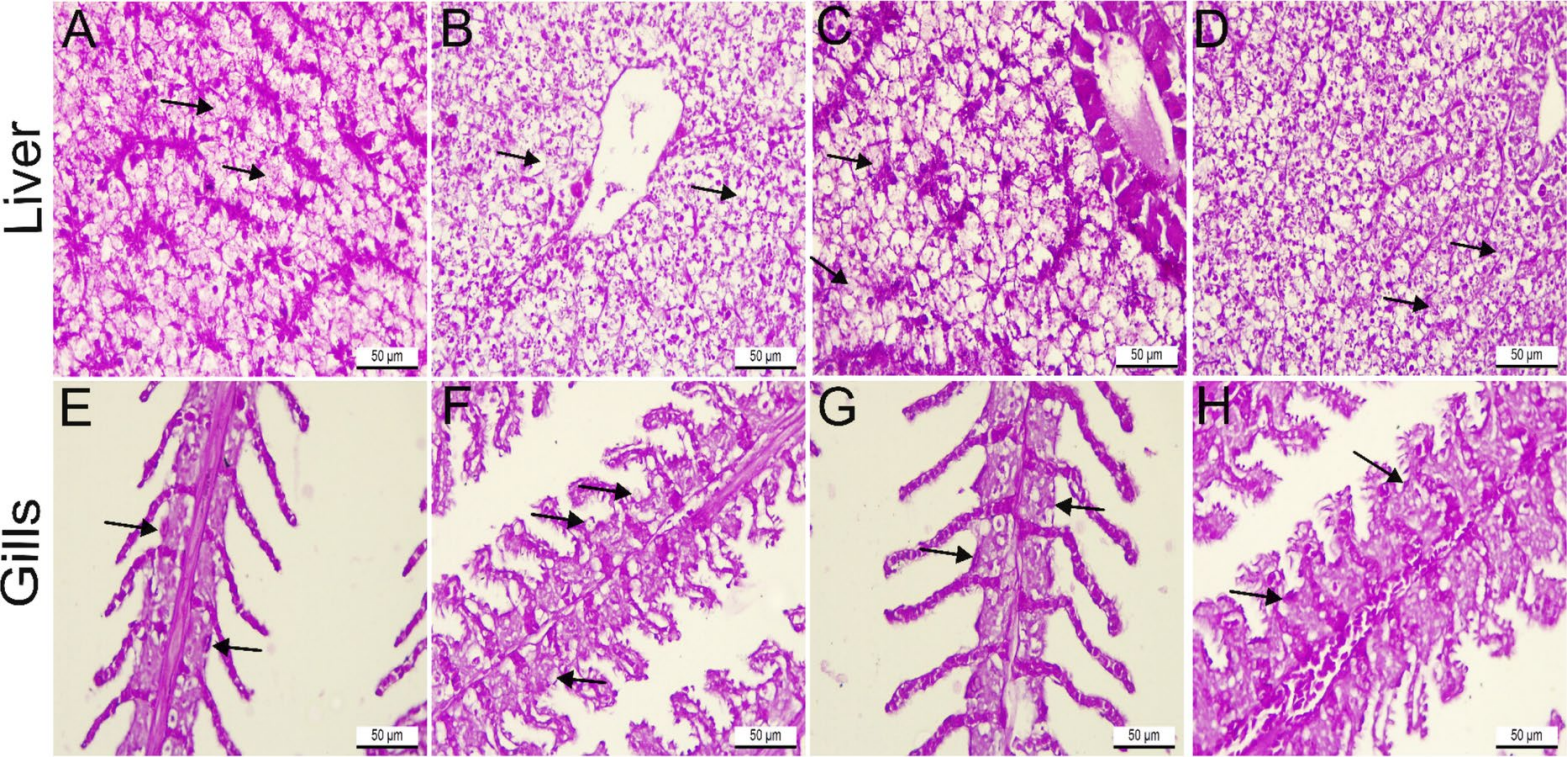
Histochemical periodic acid-Schiff-stained liver (**A–D**) and gill (**E**–**H**) sections of *O. niloticus*. × 400. Control fish in group I showed positive reaction (back arrows) in **A** hepatocytes cytoplasm and **E** gill mucous cells. Fish challenged with *E. faecalis* in group II had mild histochemical reaction (black arrows) in **B** hepatocytes cytoplasm and **F** gill mucous cells. Fish exposed to thyme-AgNPs in group III revealed positive reactions (black arrows) in **C** hepatocytes cytoplasm and **G** gill mucous cells. *E. faecalis* challenged fish and exposed to thyme-AgNPs in group IV showed moderate histochemical reaction (back arrows) in **D** hepatocytes cytoplasm and **H** gill mucous cells

**Fig. 9:**
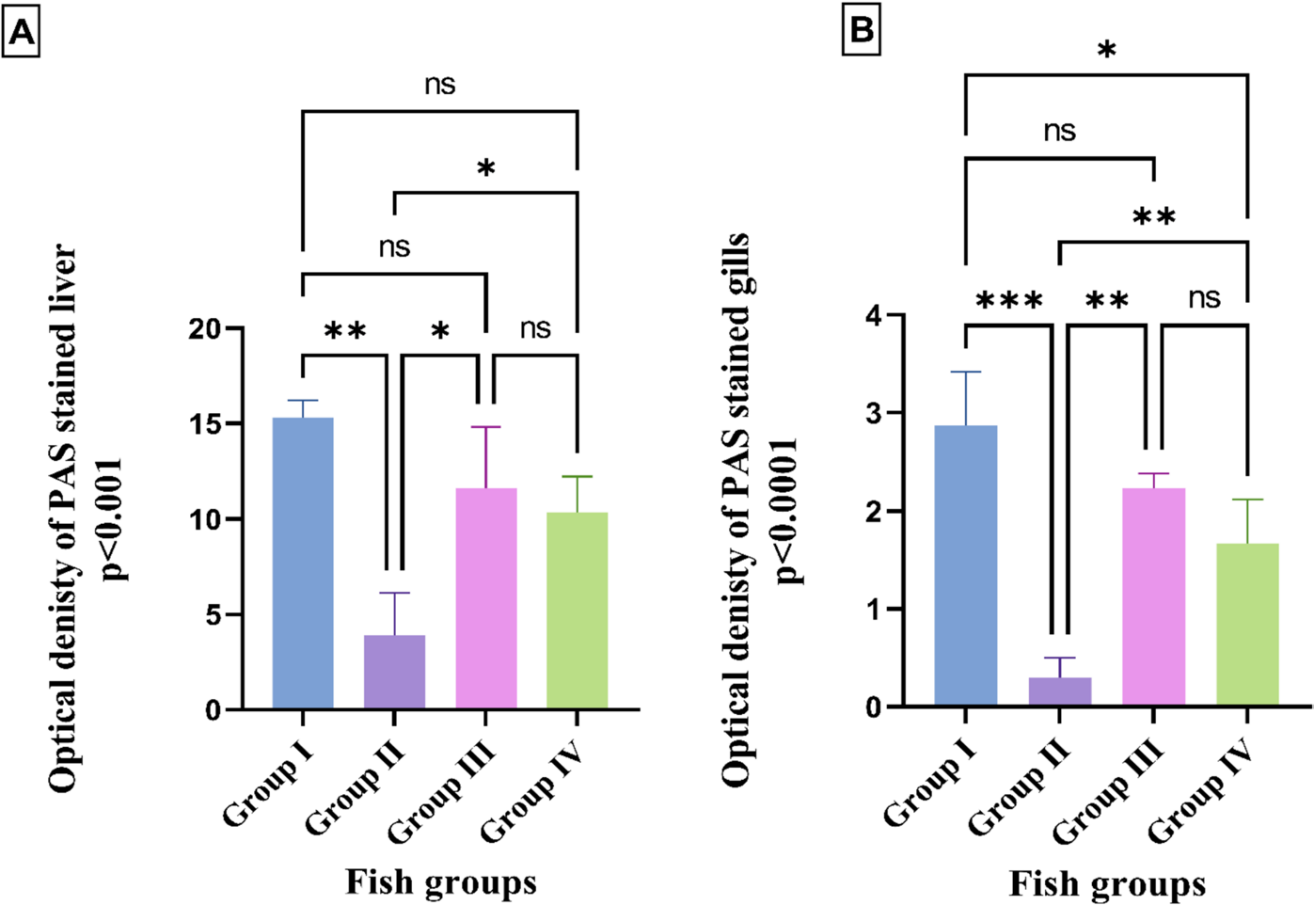
**A**–**B** The effect of *E. faecalis* and/or thyme-AgNPs on the optical density of periodic acid-Schiff positive materials within hepatocytes cytoplasm and gill mucous cells. The results were expressed as mean ± SE.* p*-value ≤ 0.001. Group I: control; group II: fish group challenged with *E.faecalis*; group III: thyme-AgNPs exposed group; group IV: fish group challenged with *E. faecalis* and exposed to thyme-AgNPs

Periodic acid-Schiff-stained branchial and hepatic sections of *O. niloticus* fish in the control group exhibited a positive histochemical reaction with normal distribution of neutral mucopolysaccharides in gill mucous cells and hepatocyte cytoplasm (Fig. 8A and E). However, the tissue of fish challenged with *E. faecalis* in group II revealed a mild histochemical reaction, with significant depletion of neutral mucopolysaccharides in hepatocytes and gill mucous cells, at 3.9 and 0.3, respectively, compared to the control group (Figs. 8B and F, and 9A and B). *O. niloticus* that was exposed to thyme-AgNPs in group III displayed a positive histochemical reaction with a non-significantly lower distribution of neutral mucopolysaccharides compared to the control fish (Figs. 8C and G, and 9A and B). Meanwhile, *E. faecalis-*challenged fish co-exposed to thyme-AgNPs in group IV displayed moderate histochemical reaction with significant restoration of neutral mucopolysaccharides in hepatocytes and gill mucous cells by 10.3 and 1.6, respectively, compared to the *E. faecalis-*challenged group (Figs. 8D and H, and 9A and B).

## Discussion

Over the past few years, controlling fish diseases against devastating pathogens has faced significant challenges due to antimicrobial resistance (Abdel-moneam et al. 2025b). Thus, searching for an effective alternative to traditional antibiotics is a necessary solution to address this issue naturally and effectively (Tural et al. 2019). Nanotechnology using green-synthesized nanoparticles has been introduced in aquaculture as a recent and safe approach to combat the emergence of microbial resistance (Abdelhameed et al. 2022). To the best of our knowledge, this is likely the first study to investigate the antibacterial activity of thyme-synthesized AgNPs against the MDR *E. faecalis* bacteria using *O. niloticus* as an experimental model.

As an emerging fish pathogen, *E. faecalis* has garnered significant attention due to its role in severe fish disease outbreaks that have had a profound and adverse impact on aquaculture practices worldwide, resulting in substantial financial losses (Elbahnaswy et al. 2023). *E. faecalis* was believed to be one of the causes of hemorrhagic septicemia in fish with a high mortality rate (Uma et al. 2022). It was isolated from many cultured fish species worldwide, such as Nile tilapia (*Oreochromis niloticus*), African catfish (*Clarias gariepinus*), meager (*Argyrosomus regius*), zebrafish (*Danio rerio*), and koi fish (*Anabas scandens*) (Uma et al. 2020; Abdelsalam et al. 2021; Nada et al. 2022; Akter et al. 2023; Rana et al. 2023; Aboyadak and Ali 2024).

In the current study, the isolated *E. faecalis* was identified biochemically and genotypically confirmed using *16S rRNA* gene sequence alignment (PQ084785). The phylogenetic analysis confirmed that the sequenced isolate belongs to the same genus as *Enterococcus*.

As evidenced by the antibiogram analysis, the *E. faecalis* isolate presented resistance to a variety of antibiotic classes, including macrolides (erythromycin and clindamycin), aminoglycosides (gentamicin and streptomycin), and tetracyclines (tetracycline), so it was classified as a multidrug-resistant (MDR) strain (Arias and Murray 2012; Samani et al. 2021). The pathogenic, MDR bacteria have evolved and modified their defense strategies against antibiotics over time by reducing the permeability of their outer membranes, forming protective biofilms, and utilizing adherence and invasion virulence factors that prevent antibiotics from entering cells. As a result, this can impede the efficacy of these antimicrobial agents against MDR bacteria (Hossain et al. 2016; Khalefa et al. 2025).

AgNPs have been recently incorporated as a promising alternative in the fight against a diverse array of Gram-negative and Gram-positive MDR fish pathogens (Kailasa et al. 2019; Vazquez-Munoz and Lopez-Ribot 2020). In the current study, AgNPs were prepared using thyme essential oil as a reducing and stabilizing agent. This enhances the antibacterial efficacy of the prepared nanoparticles, as they secrete signal-dissolving enzymes that denature and interfere with bacterial signaling molecules by hindering and blocking them (Akshaya et al. 2022; Yousef et al. 2022). Moreover, it was suggested that AgNPs produced using a biological method are smaller in size and more active compared to chemically synthesized ones, thus allowing them to penetrate bacterial cell membranes more effectively and disrupt various metabolic functions, causing cell lysis and death. This explains why they have the most effective antibacterial properties (Chauhan et al. 2013; Gurunathan 2019).

The in vitro antibacterial activity of thyme-AgNPs against the tested MDR *E. faecalis* isolate was assessed by the formation of a clear circular inhibition zone around the wells impregnated with thyme-AgNPs. The AgNPs’ antimicrobial effect was concentration dependent; it was strongest at higher concentrations (1000, 500, 250, 125, and 62 µg/mL), with zone diameters of 22, 20, 18, 16, and 14 mm, respectively, but no zones were recorded at the lowest concentration (< 62.5). The complete growth inhibition of *E. faecalis*, verified by this isolate after incubation with AgNPs in MH broth at 62.5 µg/mL (MBC) and 31.5 µg/mL (MIC), with an MBC/MIC ratio of two, confirmed the bactericidal potential of thyme-AgNPs. In the same vein, Hamida et al. (2020b) and Crisan et al. (2024) reported that AgNPs exhibited inhibition zones against some Gram-positive bacteria, including *S. aureus* (MRSA) and *E. faecalis*.

Throughout the 15-day in vivo experimental duration*,* no mortalities or atypical clinical findings were observed in all fish exposed groups except for the fish in group II that was experimentally challenged with *E. faecalis*, where fish exhibited skin darkness, abnormal swimming behavior with poor reflexes, generalized septicemia, internal hemorrhages, and a high mortality rate similar to the reports by Elgohary et al. (2021) and Hassan et al. (2022). This finding may be related to the virulence factors of *E. faecalis*, including hemolysin, cytolysin, gelatinase, surface proteins, and lipoteichoic acid, which are responsible for disease pathogenesis and play a vital role in inflammatory responses (El-Refaey 2013; Park et al. 2013; Mishra et al. 2017).

Conversely, no clinical signs were observed in the control and thyme-AgNPs-exposed groups (groups III and IV). Reduced mortalities were observed in group IV, which was challenged with *E. faecalis* and simultaneously exposed to thyme-AgNPs. This indicated the antibacterial and immune-modulatory effects of thyme-AgNPs, as confirmed in the present study, which in turn enhanced the general health status of the fish and hindered the progression of the disease, resulting in minimized mortalities (Halkai et al. 2018).

Serum IgM is the predominant immunoglobulin class in teleost fish and is considered a key indicator of the humoral immune system’s early reaction to pathogens and foreign substances (Salinas et al. 2021). In our study, the significant reduction in IgM levels in the *E. faecalis*-challenged group (group II) could imply an immunosuppressive effect of the current bacterial infection on the immune system response, resulting from IgM depletion or redistribution (Boulware and Mielke 2018). Conversely, the IgM levels in fish in group IV were significantly elevated, suggesting that nanoparticles have immunomodulatory effects by stimulating IgM production, in agreement with El-Houseiny et al. (2021). As mentioned by Bai et al. (2016) and Sun et al. (2020), co-treatment with nanoparticles enhances the immune response by improving antigen presentation, processing, and activating immune cells, even in the presence of an infection. In line with AL-Rhman et al. 2016), thyme-AgNPs-exposed fish in group III revealed a notable increase in serum IgM levels, indicating the potential immune-boosting properties of nanoparticles, which act as an antigenic stimulus and activate the immune response (Patel et al. 2020).

In aquatic organisms, oxidative stress biomarkers and antioxidant defenses serve as valuable diagnostic and prognostic tools (Abdel-Radi et al. 2022; Shija et al. 2023). Several studies have reported an association between bacterial infection and oxidative stress in fish (Baldissera et al. 2018; Said et al. 2023; Gamal et al. 2024).

In the current study, an increase in oxidant indicators (MDA) was observed, accompanied by significant reductions in antioxidant indicators (GSH, CAT, and SOD) in the gills and liver of fish challenged with *E. faecalis*. Oxidative stress is a state of oxidant-antioxidant imbalance that stimulates the initial inflammatory response, leading to the production of additional reactive oxygen species (ROS) and, consequently, further tissue damage (Yang et al. 2023; Khalefa et al. 2024). Excessive ROS production induces damage to cellular biomolecules, including lipids, leading to lipid peroxidation, as indicated by the presence of MDA (Souza et al. 2017; Morselli et al. 2020). In agreement with the current results, Zahran et al. (2019) and Chen et al. (2020) recorded elevated levels of MDA and a depressed antioxidant mechanism in cases of *E. faecalis* and *A. hydrophila* bacterial infections*.* Moreover*,* Bortoletti et al. (2022) and Schumann et al. (2023) reported that excessive ROS production in response to infectious diseases could lead to increased lipid peroxidation and a weakening of the antioxidant defense system over time.

A key component of fish antioxidant defense is GSH, which can conjugate with oxidative stress byproducts, reducing their toxicity and promoting their elimination from the host cell (Wang et al. 2021). The marked oxidative damage observed in the gills and liver of fish challenged with *E. faecalis* in group II may be attributed to GSH depletion in response to scavenging excess ROS-induced oxidative stress (Genchi et al. 2021). Similarly, Vinosha et al. (2020) and Junior and Baldisserotto (2020) reported a significant reduction in hepatic GSH content in cases of *Aeromonas* bacterial infection, making fish more susceptible to oxidative damage.

The ROS levels are controlled not only by their production but also by their elimination. In this sense, aquatic organisms possess multilevel and complex antioxidative defenses that manage ROS formation and elimination, thereby minimizing ROS-related toxicity (Lushchak 2016). CAT and SOD antioxidant enzymes are the first line of defense against ROS, as they detoxify ROS and counteract their damaging effects. There is a direct correlation between CAT and SOD levels, where the SOD enzyme converts the superoxide anion (O_2_^−^) to hydrogen peroxide (H_2_O_2_), and the CAT enzyme converts the resulting H_2_O_2_ to water (H_2_O) and oxygen (O_2_) (Halliwell 2012). The recorded downregulation in CAT and SOD levels is similar to that documented by Kurhalyuk and Tkachenko (2011), Souza et al. (2019), El-Habashi et al. (2019), and El-Houseiny et al. (2021) in the case of bacterial infection. This could be related to the disease pathophysiology and the inability of the antioxidant defense system to protect host cells against ROS in case of *E. faecalis* infection (Baldissera et al. 2018).

Herein, significant upregulation in the expression of inflammatory cytokine genes (*TNF-α* and *IL-1β*) was observed in the gills and liver of *E. faecalis*-challenged fish in group II. Like mammals, the primary defense mechanism against infectious diseases in aquatic animals is the innate immune system, which is mainly regulated through cytokines (Sims and Smith 2010). *TNF-α* and *IL-1β* are pro-inflammatory cytokines involved in the immune-mediated response against various fish pathogens, initiating the early immune response (Sherif et al. 2025; Mohdali et al. 2025). *TNF-α* can activate chemokine expression in local tissue cells, such as endothelial cells, and exhibits additional pro-apoptotic activities (Praveen et al. 2006; Roca et al. 2008). *IL-1β* coordinates fish responses to infections by activating lymphocytes and inducing the release of other cytokines (Zou and Secombes 2016). The current results are supported by those of Reda et al. (2024), which demonstrated that *Shewanella* bacterial infection promotes the upregulation of *TNF-α* and *IL-1β* levels. Similarly, a study conducted by El Gamal et al. (2023) revealed that the immune genes *TNF-α* and *IL-1β* were significantly elevated in the gills following infection with the fungal species *Saprolegnia parasitica*. In addition, El-Houseiny et al. (2021), Baloch et al. (2022), Abou-Okada et al. (2023), and Alaryani et al. (2024) recorded elevated levels of inflammatory cytokines (*TNF-α* and *IL-1β*) in infected fish with *A. hydrophila, V. alginolyticus, E. faecalis,* and cyprinid herpes virus infection. In contrast, Zahran et al. (2019) reported that *E. faecalis* experimental infection demonstrated no statistically significant change in *IL-8* and *TNF-α* mRNA transcriptional levels. These differences may be attributed to variations in the causative agent, the severity of the infection, and the fish’s immune status.

The intervention of thyme-AgNPs to the challenged fish with *E. faecalis* in group IV revealed a reverse modulation in the levels of oxidant/antioxidant parameters and inflammatory response through significantly reduced levels of MDA, increased GSH levels, and upregulation of antioxidant-related genes (*CAT* and *SOD*), along with substantial downregulation of immune-related genes transcription levels (*TNF-α* and *IL-1β).* This confirmed that thyme-AgNPs potentially ameliorate *E. faecalis-*induced oxidative stress and inflammation. These properties may be attributed to the presence of functional groups, such as thymus polyphenols, on the surface of AgNPs, which could be responsible for their antioxidant and ROS scavenging properties. The antioxidant and anti-inflammatory properties of green-synthesized AgNPs have been reported in several studies conducted by Behravan et al. (2019), Keshari et al. (2020), Nandhini and Sheeba (2020), Bidaki et al. (2024), and Singh et al. (2024).

Histopathological findings of *O. niloticus* fish challenged with *E. faecalis* in group II revealed various alterations in both branchial and hepatic tissue structures compared to the control fish, which did not exhibit any adverse pathological effects. These findings align with the results of Abu-Elala et al. (2020) and Alaryani et al. (2024), which indicate that *E. faecalis*-infected tilapia exhibit congested venous sinuses with epithelial hyperplasia of the gill filaments, accompanied by liver vacuolar degeneration and congested hepatic sinusoids. These observed alterations were considered tissue responses to toxins and extracellular products in cases of bacterial infections (Pal et al. 2012; A’yunin et al. 2020). The observed vacuolar degeneration may be considered a cellular defense mechanism against hepatocytic injury (Mollendorff 1973). Javed et al. (2016) reported that hemorrhage may occur due to the congestion of blood vessels caused by increased blood flow in response to exposure to bacterial toxins.

However, the exposure of *E. faecalis-*challenged *O. niloticus* to thyme-AgNPs in group IV resulted in marked improvement in the histoarchitecture of both gills and liver tissue. This may be attributed to the powerful antioxidant and antibacterial effects of thyme-AgNPs (Behravan et al. 2019; Abdellatif et al. 2022). Meanwhile, *O. niloticus* exposed to thyme-AgNPs in group III exhibited a few changes in gill and liver tissue structure compared to control fish.

Histochemistry serves as a valuable cellular biomarker for assessing the impact of environmental pollution and bacterial exposure (Yancheva et al. 2019). Periodic acid-Schiff (PAS) is a special indicator stain that primarily detects polysaccharide and mucopolysaccharide components, such as glycogen and mucin, in tissues, producing a magenta-colored reaction (Shedge et al. 2020; Giardino et al. 2023). Interestingly, the present results demonstrated that *O. niloticus* challenged with *E. faecalis* in group II revealed a significant depletion of neutral mucopolysaccharide in both mucous cells in the gills and hepatocytes in the liver, characterized by a faint purple coloration, compared to the control fish. Otherwise, exposure to thyme-AgNPs in group IV resulted in the restoration of neutral mucopolysaccharide distribution, as indicated by increased purple coloration. This aligns with Rao (2006), who suggests that mucopolysaccharide depletion in fish tissues is a typical stress-related response to various toxicants, aimed at meeting the increased energy demands induced by elevated metabolic activity (Sayed et al. 2024).

## Conclusion

Building on the study outcomes, *O. niloticus* fish challenged with MDR *E. faecalis* exhibited an obvious septicemic picture*,* characterized by high mortality, immune suppression, oxidative damage, and upregulation of immune-related gene transcription levels, accompanied by histopathological and histochemical alterations. However, the intervention of thyme-AgNPs revealed an improvement in the general health status of fish by boosting IgM levels, noticeably modulating the oxidant-antioxidant balance, restoring the histological architecture with improved gene expression, and causing no toxicity to the fish. In this regard, thyme-AgNPs act as a potent and eco-friendly antibacterial agent capable of counteracting the MDR *E. faecalis*-induced pathologies in *O. niloticus* and can be used safely to control fish diseases in the coming decades. However, further studies on the antimicrobial efficacy of thyme-AgNPs against different Gram-positive and Gram-negative fish pathogens are required.

## Supplementary Information

Below is the link to the electronic supplementary material.

Supplementary file1 (DOCX 5353 KB)

## Data Availability

Data is provided within the manuscript or supplementary information files.

## References

[CR1] A’yunin Q, Budianto Andayani S, Yuwanita R (2020) Histopathological analysis of *Pangasius* sp. infected by *Edwardsiella tarda* causes Edwardsiellosis disease. IOP Conf Ser Earth Environ Sci 441:012031. 10.1088/1755-1315/441/1/012031

[CR2] Abd El Megeed OH, Mabrouk MM, Gewida AGA et al (2025) The mitigative effect of selenium nanoparticles against aluminium nanoparticle toxicity in Nile tilapia (*Oreochromis niloticus*): growth performance, antioxidant status, gene expression, and histopathological changes. Aquacult Int 33:443. 10.1007/s10499-025-02128-z

[CR3] Abd El-Hamid MI, Ibrahim SM, Eldemery F, El-Mandrawy SAM, Metwally AS, Khalifa E, Elnahriry SS, Ibrahim D (2021) Dietary cinnamaldehyde nanoemulsion boosts growth and transcriptomes of antioxidant and immune related genes to fight *Streptococcus agalactiae* infection in Nile tilapia (*Oreochromis niloticus*). Fish Shellfish Immunol 113:96–105. 10.1016/j.fsi.2021.03.021 33826939 10.1016/j.fsi.2021.03.021

[CR4] Abdel-moneam DA, Khalefa HS, Rashad MM, Ali GE, Bashir DW, Noshy PA, Mahmoud SB (2025a) Dietary carvacrol essential oil alleviates thallium nitrate-induced toxicity in *Oreochromis niloticus*: growth, hepatorenal, oxidative stress, gene expression, and histopathological study. Aquaculture 599:742175. 10.1016/j.aquaculture.2025.742175

[CR5] Abdel-moneam DA, Khalefa HS, Shaalan M, Elshafiee EA, Ahmed ZS (2025b) Assessment of the role of wild waterfowl as potential vectors of *Aeromonas hydrophila* and its cross-transmission to Qarun Lake’s aquatic environment, considering the altered water quality parameters. Biol Bull 52:13. © Pleiades Publishing, Inc., 2025. ISSN 1062–3590

[CR6] Abdel-Radi S, Rashad MM, Ali GE, Eissa AE, Abdelsalam M, Abou-Okada M (2022) Molecular characterization and phylogenetic analysis of parasitic copepoda; *Ergasilus sieboldi* isolated from cultured gilthead sea bream (*Sparus aurata*) in Egypt, associated with analysis of oxidative stress biomarkers. J Parasit Dis 46(4):1080–1089 36457775 10.1007/s12639-022-01531-0 PMC9606149

[CR7] Abdelhameed RM, Abu-Elghait M, El-Shahat M (2022) Engineering titanium-organic framework decorated silver molybdate and silver vanadate as antimicrobial, anticancer agents, and photo-induced hydroxylation reactions. J Photochem Photobiol Chem 423:113572. 10.1016/j.jphotochem.2021.113572

[CR8] Abdelkhalek S, Attia MM, Ibrahim MA et al (2024) Alterations in histopathology and stress-associated gene expression induced by infection with *Prohemistomum vivax* encysted metacercariae in Nile tilapia. Aquacult Int 32:5107–5124. 10.1007/s10499-024-01418-2

[CR9] Abdellatif AA, Alhathloul SS, Aljohani AS, Maswadeh H, Abdallah EM, Hamid Musa K, El Hamd MA (2022) Green synthesis of silver nanoparticles incorporated aromatherapies utilized for their antioxidant and antimicrobial activities against some clinical bacterial isolates. Bioinorg Chem Appl 2432758:14. 10.1155/2022/2432758 10.1155/2022/2432758 PMC9017581 35449714

[CR10] Abdelsalam M, Ewiss MZ, Khalefa HS, Mahmoud MA, Elgendy MY, Abdel-Moneam DA (2021) Coinfections of *Aeromonas* spp., *Enterococcus faecalis*, and *Vibrio alginolyticus* isolated from farmed Nile tilapia and African catfish in Egypt, with an emphasis on poor water quality. Microb Pathog 160:105213 34582943 10.1016/j.micpath.2021.105213

[CR11] Abou-Okada M, Rashad MM, Ali GE, Abdel-Radi S, Hassan A (2023) Oxidative stress, gene expression and histopathology of cultured gilthead sea bream (*Sparus aurata*) naturally co-infected with *Ergasilus sieboldi* and *Vibrio alginolyticus*. BMC Vet Res 19(1):277 38104092 10.1186/s12917-023-03840-9 PMC10724927

[CR12] Aboyadak I, Ali N (2024) Effect of *Enterococcus faecalis* infection on some immune parameters, immune genes expression and tissue pathology of *Argyrosomus regius*. Aquacult Int 32:8249–8266. 10.1007/s10499-024-01565-6

[CR13] Abu-Elala N, Abd-Elsalam R, Abouel Karamat N (2020) Streptococcosis, lactococcosis and enterococcosis are potential threats facing cultured Nile tilapia (*Oreochomis niloticus*) production. Aquac Res 51:1–13. 10.1111/are.14760

[CR14] Ahmed SA, El-Rahman GIA, Mohammed HA, Abdo SA, Aly MY, Ghannam HE, Ibrahim RE (2025) The ability of dietary essential oils to mitigate nickel-induced growth retardation, immune-antioxidant suppression, and endoplasmic reticulum stress activation in Nile tilapia. Fish Physiol Biochem 51(2):76 40163232 10.1007/s10695-025-01482-2 PMC11958502

[CR15] Akshaya T, Aravind M, Kumar S, Baskaran D (2022) Evaluation of in-vitro antibacterial activity against gram-negative bacteria using silver nanoparticles synthesized from *Dypsis lutescens* leaf extract. J Chil Chem Soc 67:5477–5483. 10.4067/S0717-97072022000205477

[CR16] Akter T, Foysal MJ, Alam M, Ehsan R, Paul SI, Momtaz F, Siddik MAB, Tay AC Y, Fotedar R, Sanjay KG, Islam T, Rahman MM (2021) Involvement of Enterococcus species in streptococcosis of Nile tilapia in Bangladesh. Aquaculture 531:735790. ‏10.1016/j.aquaculture.2020.735790

[CR17] Akter T, Haque MN, Ehsan R (2023) Virulence and antibiotic-resistance genes in *Enterococcus faecalis* associated with streptococcosis disease in fish. Sci Rep 13:1551. 10.1038/s41598-022-25968-8 36707682 10.1038/s41598-022-25968-8 PMC9883459

[CR18] Al-Jahani GMAM (2021) Thymus vulgaris (thyme) as a natural organic matter to biosynthesis silver nanoparticles and their antibacterial efficiency. Int J Pharm Res Allied Sci 10(1):118–26. 10.51847/oBv07gPJ5C

[CR19] AL-Rhman RMA, Ibraheem SR, Israa AO (2016) The effect of silver nanoparticles on cellular and humoral immunity of mice in vivo and in vitro. Iraqi J Biotech 15:21–29

[CR20] Alaryani FS, Albaqami NM, Elbahnaswy S, Elshopakey GE, Almutairi LA, Hendam BM, Khattab MS, Abd El Megeed OH, Mathew RT, Kari ZA, Abd El-Aziz YM (2024) Effects of vitamin E and/or selenium nanoparticles on organ histology, hemato-biochemical parameters, immunity, gene expression, and growth performance in Nile tilapia challenged with Enterococcus faecalis. Aquacult Rep 39:102514. 10.1016/j.aqrep.2024.102514

[CR21] Arias CA, Murray BE (2012) The rise of the Enterococcus: beyond vancomycin resistance. Nat Rev Microbiol 10(4):266–278. 10.1038/nrmicro2761 22421879 10.1038/nrmicro2761 PMC3621121

[CR22] Ayala-Núñez NV, Villegas HL, Turrent LC, Padilla CR (2009) Silver nanoparticles toxicity and bactericidal effect against methicillin-resistant *Staphylococcus aureus*: nanoscale does matter. NanoBiotechnology 5:2–9

[CR23] Bai Y, Zhang X, Liu S (2016) Nanoparticles as immune modulators: current advances in their use in immune diseases. Front Immunol 7:400. 10.3389/fimmu.2016.00400 27752255 10.3389/fimmu.2016.00400 PMC5046094

[CR24] Baldissera MD, Souza CF, Parmeggiani B, Leipnitz G, Verdi CM, Santos RV, Stefani LM, Baldisserotto B (2018) The disturbance of antioxidant/oxidant balance in fish experimentally infected by *Aeromonas caviae*: relationship with disease pathophysiology. Microb Pathog 122:53–57 29886086 10.1016/j.micpath.2018.06.011

[CR25] Baloch AA, Abdelsalam EEE, Piaˇcková V (2022) Cytokines studied in carp (*Cyprinus carpio* L.) in response to important diseases. Fishes 7:3. 10.3390/fishes7010003

[CR26] Bancroft JD, Gamble M (2013) Theory and practice of histological techniques, 7th edn. Churchill Livingstone of Elsevier, Philadelphia, pp 172–186

[CR27] Bauer AW, Kirby WM, Sherris JC, Turck M (1966) Antibiotic susceptibility testing by a standardized single disk method. Am J Clin Pathol 45(4):493–496 5325707

[CR28] Behravan M, Panahi AH, Naghizadeh A, Ziaee M, Mahdavi R, Mirzapour A (2019) Facile green synthesis of silver nanoparticles using *Berberis vulgaris* leaf and root aqueous extract and its antibacterial activity. Int J Biol Macromol 124:148–154. 10.1016/j.ijbiomac.2018.11.101 30447360 10.1016/j.ijbiomac.2018.11.101

[CR29] Belgacem ZB, Abriouel H, Omar NB, Lucas R, Martínez-Canamero M, Gálvez A, Manai M (2010) Antimicrobial activity, safety aspects, and some technological properties of bacteriocinogenic *Enterococcus faecium* from artisanal Tunisian fermented meat. Food Control 21(4):462–470. 10.1016/j.foodcont.2009.07.007

[CR30] Bidaki MZ, Naghizadeh A, Yousefinia A, Hosseinzadeh M, Lashkari S, Mortazavi-Derazkola S, Moghanni M (2024) Environmentally friendly synthesis of silver nanoparticles using prickly pear extract and their antimicrobial and antioxidant activities. Biomass Convers Biorefin. 10.1007/s13399-023-05259-6

[CR31] Bortoletti M, Maccatrozzo L, Peruzzi S, Strand JET, Jobling M, Radaelli G, Bertotto D (2022) Dietary effects on biomarkers of growth, stress, and welfare of diploid and triploid Atlantic salmon (*Salmo salar*) during parr-smolt transformation. Aquac Rep 24:101123

[CR32] Boulware DR, Mielke LA (2018) The role of IgM in bacterial infections: mechanisms and therapeutic opportunities. J Immunol Res 1520502. 10.1155/2018/1520502

[CR33] Budhiraja N, Sharma A, Dahiya S, Parmar R, Vidyadharan V (2013) Synthesis and optical characteristics of silver nanoparticles on different substrates. Int Lett Chem Phys Astron 19:80–88. 10.56431/p-24ku7c14

[CR34] Chand K, Cao D, Fouad DE, Shah AH, Dayo AQ, Zhu K, Lakhan MN, Mehdi G, Dong S (2020) Green synthesis, characterization and photocatalytic application of silver nanoparticles synthesized by various plant extracts. Arab J Chem 13(11):8248–8261. 10.1016/j.arabjc.2020.01.009

[CR35] Chauhan R, Kumar A, Abraham J (2013) A biological approach to the synthesis of silver nanoparticles with *Streptomyces* sp JAR1 and its antimicrobial activity. Sci Pharm 81(2):607–621. 10.3797/scipharm.1302-02 23833724 10.3797/scipharm.1302-02 PMC3700086

[CR36] Chen J, Liu N, Zhang H, Zhao Y, Cao X (2020) The effects of *Aeromonas hydrophila* infection on oxidative stress, nonspecific immunity, autophagy, and apoptosis in the common carp. Dev Comp Immunol 105:103587 31875516 10.1016/j.dci.2019.103587

[CR37] CLSI (Clinical and Laboratory Standards Institute) (2012) Performance standards for antimicrobial susceptibility testing; twenty-second informational supplement (CLSI document M100-S22). Wayne, PA: CLSI

[CR38] CLSI (Clinical and Laboratory Standards Institute) (2021) performance standards for antimicrobial susceptibility testing; 31st Edition (M100-S31). Wayne, PA: CLSI 10.1128/JCM.00213-21 PMC8601225 34550809

[CR39] Crisan MC, Pandrea SL, Matros L, Mocan T, Mocan L (2024) In vitro antimicrobial activity of silver nanoparticles against selected gram-negative and gram-positive pathogens. Med Pharm Rep 97(3):280–297. 10.15386/mpr-2750 39234464 10.15386/mpr-2750 PMC11370865

[CR40] Dahdouh B, Basha O, Haggag Y, Khalil S, Tanekhy M (2020) Antibacterial effects of nano-silver suspension against some fish pathogens (in vitro). Alexandria J Vet Sci 67(2):75–81. 10.5455/ajvs.134532

[CR41] de Melo APZ, Maciel MVO, Sganzerla WG, Almeida AR, Armas RD, Machado MH, Rosa CG, Nunes MR, Bertoldi FC, Barreto PLM (2020) Antibacterial activity, morphology, and physicochemical stability of biosynthesized silver nanoparticles using thyme (*Thymus vulgaris*) essential oil. Mater Res Express 7:015087. 10.1088/2053-1591/ab6c63

[CR42] Devriese LA, Pot B, Collins MD (1993) Phenotypic identification of the genus *Enterococcus* and differentiation of phylogenetically distinct enterococcal species and species groups. J Appl Bacteriol 75(5):399–408. 10.1111/j.1365-2672.1993.tb02794.x 8300442 10.1111/j.1365-2672.1993.tb02794.x

[CR43] Dong H, Nguyen V, Le H, Sangsuriya P, Jitrakorn S, Saksmerprome V, Senapin S, Rodkhum C (2015) Naturally concurrent infections of bacterial and viral pathogens in disease outbreaks in cultured Nile tilapia (*Oreochromis niloticus*) farms. Aquaculture 448:427–435. 10.1016/j.aquaculture.2015.06.027

[CR44] Dorojan OG, Cristea V, Creţu M, Coadă MT, Dediu L, Grecu IR (2015) Effect of thyme (*Thymus vulgaris*) and vitamin E on growth performance and body composition of *Acipenser stellatus* juveniles. Aquacult Aquarium Conserv Legislat Int J Bioflux Soc 8(2):195–202

[CR45] Dube E (2024) Antibacterial activity of engineered nanoparticles against fish pathogens. Aquac Rep 37:102240. 10.1016/j.aqrep.2024.102240

[CR46] Eden PA, Schmidt TM, Blakemore RP, Pace NR (1991) Phylogenetic analysis of *Aquaspirillum magnetotacticum* using polymerase chain reaction-amplified 16S rRNA-specific DNA. Int J Syst Bacteriol 41(2):324–325. 10.1099/00207713-41-2-324 1854644 10.1099/00207713-41-2-324

[CR47] El Gamal SA, Adawy RS, Zaki VH, Zahran E (2023) Host–pathogen interaction unveiled by immune, oxidative stress, and cytokine expression analysis to experimental *Saprolegnia parasitica* infection in Nile tilapia. Sci Rep 13(1):9888. 10.1038/s41598-023-36892-w 37337042 10.1038/s41598-023-36892-w PMC10279727

[CR48] El-Habashi N, Fadl SE, Farag HF, Gad DM, Elsadany AY, El Gohary MS (2019) Effect of using *Spirulina* and *Chlorella* as feed additives for elevating immunity status of Nile tilapia experimentally infected with *Aeromonas hydrophila*. Aquac Res 50:2769–2781

[CR49] El-Houseiny W, Mansour MF, Mohamed WAM, Al-Gabri NA, El-Sayed AA, Altohamy DE, Ibrahim RE (2021) Silver nanoparticles mitigate *Aeromonas hydrophila*-induced immune suppression, oxidative stress, and apoptotic and genotoxic effects in *Oreochromis niloticus*. Aquaculture 535:736430

[CR50] El-Refaey A (2013) Studies on major bacterial diseases affecting fish; Tilapia *Oreochromis niloticus*, Catfish, *Clarias gariepinus* and mullets in Port Said, Egypt with special references to its pathological alterations. Researcher 5(2):5–14

[CR51] Elbahnaswy S, Elshopakey GE, Shakweer MS, Eldessouki EA, Abdelwarith AA, Younis EM, Davies SJ, El-Son MA (2023) Bacterial co-infection as a potential threat to farmed flathead grey mullet (*Mugil cephalus*): phenotypic and molecular diagnosis, histopathology, immunity response, and in vitro antibacterial evaluation. Fishes 8(7):357. 10.3390/fishes8070357

[CR52] Elgendy M, Shaalan M, Abdelsalam M, Eissa A, El-Adawy M, Seida A (2022) Antibacterial activity of silver nanoparticles against antibiotic-resistant *Aeromonas veronii* infections in Nile tilapia, *Oreochromis niloticus* (L.), in vitro and in vivo assay. Aquac Res 53:901–920. 10.1111/are.15632

[CR53] Elgohary I, Eissa AE, Fadel NG, Abd Elatief JI, Mahmoud AM (2021) Bacteriological, molecular and pathological studies on some gram-positive bacteria; *Aerococcus viridans* and; *Enterococcus faecalis* affecting *Oreochromis niloticus* in some Egyptian fish farms. Aquacult Res 52:2220–2232

[CR54] Fakharzadeh S, Hafizi M, Baghaei MA, Etesami M, Khayamzadeh M, Kalanaky S, Akbari ME, Nazaran MH (2020) Using nanochelating technology for biofortification and yield increase in rice. Sci Rep 10(1):1–9. 4351. 10.1038/s41598-020-60189-x 10.1038/s41598-020-60189-x PMC7062768 32152326

[CR55] FAO (2024) The State of World Fisheries and Aquaculture 2024. Blue Transformation in Action, Rome

[CR56] Finney DJ (1971) Probit Analysis, 3rd edn. Cambridge University Press, Cambridge

[CR57] Gamal A, Abdel-moneam DA, Morsi AS, Malak NML, Ali AM, Khalefa HS (2024) In-vitro and in-vivo assessment of the bactericidal potential of peracetic acid and hydrogen peroxide disinfectants against *A. hydrophila* infection in Nile tilapia and their effect on water quality indices and fish stress biomarkers. Sci Rep 14:25715. 10.1038/s41598-024-76036-2 39468161 10.1038/s41598-024-76036-2 PMC11519942

[CR58] Genchi G, Carocci A, Lauria G, Sinicropi MS, Catalano A (2021) Thallium use, toxicity, and detoxification therapy: an overview. Appl Sci 11(18):8322. 10.3390/app11188322

[CR59] Giardino L, Generali L, Del Fabbro M, Tartaglia GM, Bidossi A, Savadori P (2023) Detection of bacteria in dental samples using the periodic acid-Schiff (PAS) histological stain. Micron 172:103498 37295188 10.1016/j.micron.2023.103498

[CR60] Gurunathan S (2019) Rapid biological synthesis of silver nanoparticles and their enhanced antibacterial effects against *Escherichia fergusonii* and *Streptococcus mutans*. Arab J Chem 12(2):168–180. 10.1016/j.arabjc.2014.11.014

[CR61] Halkai KR, Mudda JA, Shivanna V, Rathod V, Halkai R (2018) Antibacterial efficacy of biosynthesized silver nanoparticles against *Enterococcus faecalis* biofilm: an *in vitro* study. Contemp Clin Dent 9(2):237–241. 10.4103/ccd.ccd_828_17 29875567 10.4103/ccd.ccd_828_17 PMC5968689

[CR62] Halliwell B (2012) Free radicals and antioxidants: updating a personal view. Nutr Rev 70(5):257–265. 10.1111/j.1753-4887.2012.00476.x 22537212 10.1111/j.1753-4887.2012.00476.x

[CR63] Hamida RS, Ali MA, Goda DA, Al-Zaban MI (2020a) Lethal mechanisms of *Nostoc*-synthesized silver nanoparticles against different pathogenic bacteria. Int J Nanomed 15:10499–10517. 10.2147/IJN.S289243 10.2147/IJN.S289243 PMC7778443 33402822

[CR64] Hamida RS, Ali MA, Goda DA, Khalil MI, Al-Zaban MI (2020b) Novel biogenic silver nanoparticle-induced reactive oxygen species inhibit the biofilm formation and virulence activities of methicillin-resistant *Staphylococcus aureus* (MRSA) strain. Front Bioeng Biotechnol 8:433. 10.3389/fbioe.2020.00433 32548095 10.3389/fbioe.2020.00433 PMC7270459

[CR65] Hammerum AM, Lester CH, Heuer OE (2010) Antimicrobial-resistant enterococci in animals and meat: a human health hazard. Foodborne Pathog Dis 7(10):1137–1146. 10.1089/fpd.2010.0552 20578915 10.1089/fpd.2010.0552

[CR66] Hashem YM, El-Hamid MIA, Awad NFS, Ibrahim D, Elshater NS, El-Malt RMS, Hassan WH, Abo-Shama UH, Nassan MA, El-Bahy SM (2022) Insights into growth-promoting, anti-inflammatory, immunostimulant, and antibacterial activities of Toldin CRD as a novel phytobiotic in broiler chickens experimentally infected with *Mycoplasma gallisepticum*. Poult Sci 101(11):102154. 10.1016/j.psj.2022.102154 36182847 10.1016/j.psj.2022.102154 PMC9523390

[CR67] Hassan MA, Abdel-Naeim NS, Mabrok M, Dessouki AA, Hassan AM (2022) Isolation and identification of *Enterococcus faecalis* from cultured *Oreochromis niloticus* and *Mugil cephalus* with a special emphasis on a possible integrated control strategy. Aquacult Res 53:5521–5535. 10.1111/are.16034

[CR68] Hossain MI, Habib MA, Ahmed N (2016) A study on antibacterial activity of silver nanoparticles. J Teach Assoc RMC Rajshahi 29(2):37–41. 10.3329/taj.v29i2.39106

[CR69] Hussain Z, Jahangeer M, Sarwar A, Ullah N, Aziz T, Alharbi M, Alshammari A, Alasmari AF (2023) Synthesis and characterization of silver nanoparticles mediated by the *mentha piperita* leaves extract and exploration of its antimicrobial activities. J Chil Chem Soc. 10.4067/s0717-97072023000205865

[CR70] Ijaz I, Gilani E, Nazir A, Bukhari A (2020) Detail review on chemical, physical and green synthesis, classification, characterizations and applications of nanoparticles. Green Chem Lett Rev 13(3):223–245. 10.1080/17518253.2020.1802517

[CR71] Javed M, Ahmad I, Usmani N, Ahmad M (2016) Studies on biomarkers of oxidative stress and associated genotoxicity and histopathology in *Channa punctatus* from heavy metal polluted canal. Chemosphere 151:210–219. 10.1016/j.chemosphere.2016.02.080 26943742 10.1016/j.chemosphere.2016.02.080

[CR72] Junior GB, Baldisserotto B (2020) Fish infections associated with the genus Aeromonas: a review of the effects on oxidative status . J Appl Microbiol 131:1083—1101. © 2020 The Society for Applied Microbiology 10.1111/jam.14986 33382188

[CR73] Kailasa SK, Park TJ, Rohit JV, Koduru JR (2019) Chapter 14 -Antimicrobial activity of silver nanoparticles. Nanoparticles in Pharmacotherapy 461-484. William Andrew Publishing. 10.1016/B978-0-12-816504-1.00009-0

[CR74] Kanwal Z, Raza MA, Manzoor F, Arshad M, Rashid F, Riaz S, Pervaiz S, Naseem S (2019) In vivo anti-proliferative activity of silver nanoparticles against *Pseudomonas aeruginosa* in freshwater *Labeo rohita*. Appl Nanosci 9. 10.1007/s13204-019-01053-x

[CR75] Keshari AK, Srivastava R, Singh P, Yadav VB, Nath G (2020) Antioxidant and antibacterial activity of silver nanoparticles synthesized by *Cestrum nocturnum*. J Ayurveda Integr Med 11(1):37–44. 10.1016/j.jaim.2017.11.003 30120058 10.1016/j.jaim.2017.11.003 PMC7125370

[CR76] Khalefa HS, Abdel-Moneam DA, Ismael E, Waziry MFF, Ali MSG, Zaki MM (2021) The effect of alterations in water quality parameters on the occurrence of bacterial diseases in different aquatic environments. Adv Anim Vet Sci 9(12):2084–2094. 10.17582/journal.aavs/2021/9.12.2084.2094

[CR77] Khalefa HS, AbuBakr HO, Aljuaydi SH, Kotp YH, Al-Mokaddem AK, Abdel-moneam DA (2024) Aquatic assessment of the chelating ability of silica-stabilized magnetite nanocomposite to lead nitrate toxicity with emphasis to their impact on hepatorenal, oxidative stress, genotoxicity, histopathological, and bioaccumulation parameters in *Oreochromis niloticus* and *Clarias gariepinus*. BMC Vet Res 20:262. 10.1186/s12917-024-04094-9 38890656 10.1186/s12917-024-04094-9 PMC11184684

[CR78] Khalefa HS, Arafa AA, Hamza D (2025) Emerging biofilm formation and disinfectant susceptibility of ESBL-producing *Klebsiella pneumoniae*. Sci Rep 15:1599. 10.1038/s41598-024-84149-x 39794383 10.1038/s41598-024-84149-x PMC11724021

[CR79] Khan HA, Ahmad A, Mehboob R (2015) Nosocomial infections and their control strategies. Asian Pac J Trop Biomed 5(7):509–514. 10.1016/j.apjtb.2015.05.001

[CR80] Kumar N, Brahmchari RK, Bhushan S, Thorat ST, Kumar P, Chandan NK, Kumar M, Singh NP (2019) Synergistic effect of dietary selenium nanoparticles and riboflavin on the enhanced thermal efficiency of fish against multiple stress factors. J Therm Biol 85:102417. 10.1016/j.jtherbio.2019.102417 31657758 10.1016/j.jtherbio.2019.102417

[CR81] Kumar S, Stecher G, Li M, Knyaz C, Tamura K (2018) MEGA X: molecular evolutionary genetics analysis across computing platforms. Mol Biol Evol 35(6):1547–1549. 10.1093/molbev/msy096 29722887 10.1093/molbev/msy096 PMC5967553

[CR82] Kurhalyuk N, Tkachenko H (2011) Induction of oxidative stress and antioxidant defenses in the livers of sea trout, *Salmo trutta* L., with ulcerative dermal necrosis. Arch Pol Fish 19(2011):229–240

[CR83] Livak KJ, Schmittgen TD (2001) Analysis of relative gene expression data using real- time quantitative PCR and the 2(-delta delta C (T)). Method Nat Methods 25:402–408. 10.1006/meth.2001.1262 10.1006/meth.2001.1262 11846609

[CR84] Lushchak VI (2016) Contaminant-induced oxidative stress in fish: a mechanistic approach. Fish Physiol Biochem 42:711–747 26607273 10.1007/s10695-015-0171-5

[CR85] Magiorakos AP, Srinivasan A, Carey RB, Carmeli Y, Falagas ME, Giske CG, Harbarth S, Hindler JF, Kahlmeter G, Olsson-Liljequist B, Paterson DL, Rice LB, Stelling J, Struelens MJ, Vatopoulos A, Weber JT, Monnet DL (2012) Multidrug-resistant, extensively drug-resistant and pandrug-resistant bacteria: an international expert proposal for interim standard definitions for acquired resistance. Clin Microbiol Infect 18(3):268–281. 10.1111/j.1469-0691.2011.03570.x 21793988 10.1111/j.1469-0691.2011.03570.x

[CR86] Mansour AT, Allam BW, Srour TM, Omar EA, Nour AAM, Khalil HS (2021) The feasibility of monoculture and polyculture of striped catfish and Nile tilapia in different proportions and their effects on growth performance, productivity, and financial revenue. J Mar Sci Eng 9:586. 10.3390/jmse9060586

[CR87] Maulu S, Hasimuna OJ, Mphande J (2021) Prevention and control of streptococcosis in tilapia culture: a systematic review. J Aquat Anim Health 33(3):162–177. 10.1002/aah.10132 34121243 10.1002/aah.10132

[CR88] Mishra A, Nam GH, Gim JA, Lee HE, Jo A, Kim HS (2018) Current challenges of Streptococcus infection and effective molecular, cellular, and environmental control methods in aquaculture. Mol Cells 41(6):495–505. 10.14348/molcells.2018.2154 10.14348/molcells.2018.2154 PMC6030242 29754470

[CR89] Mishra RK, Saraf G, Patidar V (2017) A study for the presence of enterococcal virulence factors gelatinase, haemolysin among clinical isolates in a tertiary care hospital. Res Rev J Microbiol Virol 7(3):14–18. 10.37591/rrjomv.v7i3.5

[CR90] Mohdali GA, Mahdy OA, Abdel-moneam DA, Khalefa HS, Shaalan M, Marzouk MS (2025) A cross-sectional analysis and immunological study of parasitic infections in *Solea aegyptiaca* and *Tilapia zillii* from Qarun and Wadi El-Rayan Lakes. Egypt J Vet Sci. 10.21608/EJVS.2025.344377.2560

[CR91] Mollendroff A (1973) Cytology and cell physiology, 3rd edn. Academic press, New York, p p65

[CR92] More PR, Pandit S, Filippis AD, Franci G, Mijakovic I, Galdiero M (2023) Silver nanoparticles: bactericidal and mechanistic approach against drug resistant pathogens. Microorganisms 11(2):369. 10.3390/microorganisms11020369 36838334 10.3390/microorganisms11020369 PMC9961011

[CR93] Morselli MB, Baldissera MD, Souza CF, Reis JH, Baldisserotto B, Sousa AA, Zimmer F, Lopes DLA et al (2020) Effects of thymol supplementation on performance, mortality and branchial energetic metabolism in grass carp experimentally infected by *Aeromonas hydrophila*. Microb Pathog 139:103915 31809794 10.1016/j.micpath.2019.103915

[CR94] Nada A, Elsheshtawy H, Youssef F (2022) *Enterococcus faecalis* infection in the cultured *Clarias gariepinus* fish from Ismailia Governorate. Suez Canal Veterinary Medical Journal. SCVMJ 27:389–400. 10.21608/scvmj.2022.281534

[CR95] Nandhini S, Sheeba D (2020) Vegetable peel extract mediated synthesis of silver nanoparticles and its antimicrobial activities. Res J Chem Environ 24:39–44

[CR96] Nangare SN, Patil PO (2020) Green synthesis of silver nanoparticles: an eco-friendly approach. Nano Biomed Eng 12(4):281–296. 10.5101/nbe.v12i4.p281-296

[CR97] Nugroho RA, Hindryawati N, Aryani R, Manurung H, Sari YP, Nurhadi M, Nurti DD, Vieraldi M, Rudianto R, Prahastika W (2022) In vivo and in vitro assays using biosynthesized silver nanoparticles on *Aeromonas hydrophila*-infected *Clarias gariepinus*. J Appl Aquac 36:170–192. 10.1080/10454438.2022.2130737

[CR98] Pal S, Kokushi E, Koyama J, Uno S, Ghosh AR (2012) Histopathological alterations in gill, liver and kidney of common carp exposed to chlorpyrifos. J Environ Sci Health 47(3):180–195. 10.1080/03601234.2012.632285 10.1080/03601234.2012.632285 22375590

[CR99] Park OJ, Han JY, Baik JE, Jeon JH, Kang SS, Yun CH, Oh JW, Seo HS, Han SH (2013) Lipoteichoic acid of *Enterococcus faecalis* induces the expression of chemokines via TLR2 and PAFR signaling pathways. J Leukoc Biol 94:1275–1284. 10.1189/jlb.1012522 23964117 10.1189/jlb.1012522

[CR100] Parvekar P, Palaskar J, Metgud S, Maria R, Smita D (2020) The minimum inhibitory concentration (MIC) and minimum bactericidal concentration (MBC) of silver nanoparticles against *Staphylococcus aureus*. Biomater Investig Dent 7(1):105–109. 10.1080/26415275.2020.1796674 32939454 10.1080/26415275.2020.1796674 PMC7470068

[CR101] Patel R, Gupta R, Patel S (2020) **N**anoparticles as therapeutic agents in immunology. Int J Nanomed 15:1023–1040. 10.2147/IJN.S243531

[CR102] Praveen K, Evans DL, Jaso-Friedmann L (2006) Constitutive expression of tumor necrosis factor-alpha in cytotoxic cells of teleosts and its role in regulation of cell-mediated cytotoxicity. Mol Immunol 43:279–291. 10.1016/j.molimm.2005.01.012 16199264 10.1016/j.molimm.2005.01.012

[CR103] Rahman M, Rahman MM, Deb SC, Alam MS, Alam MJ, Islam MT (2017) Molecular identification of multiple antibiotic resistant fish pathogenic *Enterococcus faecalis* and their control by medicinal herbs. Sci Rep 7(1):3747. 10.1038/s41598-017-03673-1 28623336 10.1038/s41598-017-03673-1 PMC5473830

[CR104] Ramya JR, Ali S, Thanigai AK, Vijayalakshmi R, Gajendiran J, Gnanam S, Ramachandran K (2024) Antimicrobial efficiency against fish pathogens on the green synthesized silver nanoparticles. Microb Pathog 193:106725. 10.1016/j.micpath.2024.106725 38848933 10.1016/j.micpath.2024.106725

[CR105] Rana ML, Firdous Z, Ferdous FB, Ullah MA, Siddique MP, Rahman MT (2023) Antimicrobial resistance, biofilm formation, and virulence determinants in *Enterococcus faecalis* isolated from cultured and wild fish. Antibiotics 12(9):1375. 10.3390/antibiotics12091375 37760672 10.3390/antibiotics12091375 PMC10525749

[CR106] Rao JV (2006) Biochemical alterations in euryhaline fish, *Oreochromis mossambicus* exposed to sub-lethal concentrations of an organophosphorus insecticide, monocrotophos. Chemosphere 65:1814–1820 16730777 10.1016/j.chemosphere.2006.04.015

[CR107] Reda RM, El-Murr A, Abdel-Basset NA, Metwally MM, Ibrahim RE (2024) Infection dynamics of *Shewanella* spp. in Nile tilapia under varied water temperatures: a hematological, biochemical, antioxidant-immune analysis, and histopathological alterations. Fish Shellfish Immunol 149:109588. 10.1016/j.fsi.2024.109588 38677630 10.1016/j.fsi.2024.109588

[CR108] Rizkiantino R, Pasaribu FH, Soejoedono RD, Purnama S, Wibowo DB, Wibawan IWT (2021) Experimental infection of *Enterococcus faecalis* in red tilapia (*Oreochromis* hybrid) revealed low pathogenicity to cause streptococcosis. Open Vet J 11(2):309–318. 10.5455/OVJ.2021.v11.i2.16 34307089 10.5455/OVJ.2021.v11.i2.16 PMC8288741

[CR109] Roca FJ, Mulero I, Lopez-Munoz A, Sepulcre MP, Renshaw SA, Meseguer J, Mulero V (2008) Evolution of the inflammatory response in vertebrates: fish TNF-alpha is a powerful activator of endothelial cells but hardly activates phagocytes. J Immunol 181(7):5071–5081. 10.4049/jimmunol.181.7.5071 18802111 10.4049/jimmunol.181.7.5071

[CR110] Said AA, Reda RM, Metwally MM, Abd El-Hady HM (2023) Therapeutic efficacy of coriander (*Coriandrum sativum*) enriched diets in *Oreochromis niloticus*: effect on hepatic-renal functions, the antioxidant-immune response and resistance to *Aeromonas veronii*. Fish Physiol Biochem 49(4):687–709. 10.1007/s10695-023-01220-6 37438674 10.1007/s10695-023-01220-6 PMC10415512

[CR111] Salinas I, Fernández-Montero Á, Ding Y, Sunyer JO (2021) Mucosal immunoglobulins of teleost fish: a decade of advances. Dev Comp Immunol 121:104079. 10.1016/j.dci.2021.104079 33785432 10.1016/j.dci.2021.104079 PMC8177558

[CR112] Samani RJ, Tajbakhsh E, Momtaz H, Samani MK (2021) Prevalence of virulence genes and antibiotic resistance pattern in *Enterococcus faecalis* isolated from urinary tract infection in Shahrekord, Iran. Reports of Biochemistry and Molecular Biology 10(1):50–59. 10.52547/rbmb.10.1.50 34277868 10.52547/rbmb.10.1.50 PMC8279714

[CR113] Sayed AEDH, Hamed M, El-Aal MA et al (2024) Climate change induce the toxicity of black sand nanoparticles on catfish (*Clarias gariepinus*) using hemato hepatological biomarkers. BioNanoSci 14:5080–5093. 10.1007/s12668-024-01549-z

[CR114] Schumann S, Mozzi G, Piva E, Devigili A, Negrato E, Marion A, Santovito G (2023) Social buffering of oxidative stress and cortisol in an endemic cyprinid fish. Sci Rep 13(1):20579 37996569 10.1038/s41598-023-47926-8 PMC10667237

[CR115] Shaalan M, Saleh M, El-Mahdy M, El-Matbouli M (2016) Recent progress in applications of nanoparticles in fish medicine: a review. Nanomed Nanotechnol Biol Med 12(3):701–710 10.1016/j.nano.2015.11.005 26656532

[CR116] Sharma R, Dhillon A, Kumar D (2018) Mentha-stabilized silver nanoparticles for high-performance colorimetric detection of Al (III) in aqueous systems. Sci Rep 8:5189. 10.1038/s41598-018-23469-1 29581515 10.1038/s41598-018-23469-1 PMC5980094

[CR117] Shedge SA, Roy P, Shedge A, Doshi MA (2020) Periodic acid Schiff (PAS) staining: a useful technique for demonstration of carbohydrates. Med Legal Update 20(2):353–57. 10.37506/mlu.v20i2.1129

[CR118] Sherif AH, Eldessouki EA, Sabry NM, Ali NG (2023) The protective role of iodine and MS-222 against stress response and bacterial infections during Nile tilapia *(Oreochromis niloticus)* transportation. Aquacult Int. 10.1007/s10499-022-00984-7

[CR119] Sherif AH, Fadel A, Kasem EA et al (2025) The nanocomposite of chitosan-vitamin C modulates the expression of immune and antioxidant-related genes in Nile tilapia stressed with lead (Pb). Aquacult Int 33:136. 10.1007/s10499-024-01806-8

[CR120] Sherif AH, Zommara MA (2024) Selenium nanoparticles ameliorate adverse impacts of aflatoxin in Nile tilapia with special reference to *Streptococcus agalactiae* infection. Biol Trace Elem Res 202:4767–4777. 10.1007/s12011-023-04031-1 38147231 10.1007/s12011-023-04031-1 PMC11339097

[CR121] Shija VM, Amoah K, Cai J (2023) Effect of *bacillus* probiotics on the immunological responses of Nile tilapia (*Oreochromis niloticus*): a review. Fishes 8(7):366. 10.3390/fishes8070366

[CR122] Sims JE, Smith DE (2010) The IL-1 family: regulators of immunity. Nat Rev Immunol 10:89–102. 10.1038/nri2691 20081871 10.1038/nri2691

[CR123] Singh A, Singh V, Bharadwaj A, Gaur R, Agarwal S, Wahi N (2024) A quick, high-yield biogenic synthesis of antibacterial AgNPs (silver nanoparticles) via *Carica papaya* seeds. Nano Life 15(1):2450011. 10.1142/S1793984424500119

[CR124] Soltani M, Ghodratnema M, Ahari H, Ebrahimzadeh Mousavi HA, Atee M, Dastmalchi F, Rahmanya J (2009) The inhibitory effect of silver nanoparticles on the bacterial fish pathogens, *Streptococcus iniae*, *Lactococcus garvieae*, *Yersinia ruckeri*, and *Aeromonas hydrophila*. Iran J Vet Res 3(2):137–142

[CR125] Souza CDF, Baldissera MD, Verdi CM, Santos RC, Da Rocha MIU, da Veiga ML, da Silva AS, Baldisserotto B (2019) Oxidative stress and antioxidant responses in Nile tilapia *Oreochromis niloticus* experimentally infected by *Providencia rettgeri*. Microb Pathog 131:164–169. 10.1016/j.micpath.2019.04.007 30978428 10.1016/j.micpath.2019.04.007

[CR126] Souza CF, Baldissera MD, Guarda NS, Bollick YS, Moresco RN, Brusque ICM, Santos RCV, Baldisserotto B (2017) *Melaleuca alternifolia* essential oil nanoparticles ameliorate the hepatic antioxidant/oxidant status of silver catfish experimentally infected with *Pseudomonas aeruginosa*. Microb Pathog 108:61–65. 10.1016/j.micpath.2017.05.016 28487227 10.1016/j.micpath.2017.05.016

[CR127] Sun S, Chen X, Wu H (2020) Nanoparticles for enhancing immune responses in infection and immunotherapy. Front Immunol 11:1204. 10.3389/fimmu.2020.01204 32849490 10.3389/fimmu.2020.01204 PMC7424013

[CR128] Tariq F, Ahmed N, Afzal M, Khan MAU, Zeshan B (2020) Synthesis, characterization and antimicrobial activity of bacillus subtilis-derived silver nanoparticles against multidrug-resistant bacteria. Jundish J Microbiol 13(5). 10.5812/jjm.91934

[CR129] Tural S, Durmaz Y, Urçar E, Turhan S (2019) Antibacterial activity of thyme, laurel, rosemary and parsley essential oils against some bacterial fish pathogen. *Acta Aquatica* Turcica 15(4):440–447. 10.22392/actaquatr.549380

[CR130] Uma A, Harresh AHM, Rebecca G, Praveenraj J (2020) Multiple drug resistant *Enterococcus* spp. causes disease and mortality in zebra fish (*Danio rerio*). Indian J Anim Sci 90(1):116–119. 10.56093/ijans.v90i1.98241

[CR131] Uma A, Philominal P, Prabu E, Musthafa MS (2022) Dietary *Bougainvillea glabra* leaf meal on growth, haemato-biochemical responses and disease resistance in Nile tilapia. Oreochromis Niloticus against Enterococcus Faecalis Aquaculture 549:737806

[CR132] Vazquez-Munoz R, Lopez-Ribot JL (2020) Nanotechnology as an alternative to reduce the spread of COVID-19. Challenges 11(2):15. 10.3390/challe11020015

[CR133] Vinosha M, Palanisamy S, Anjali R, Li C, Yelithao K, Marudhupandi T, Tabarsa M, You SG et al (2020) Sulfated galactan from *Halymenia dilatata* enhance the antioxidant properties and prevents *Aeromonas hydrophila* infection in tilapia fish: in vitro and in vivo study. Int J Biol Macromol 158:569–579 32360202 10.1016/j.ijbiomac.2020.04.212

[CR134] Wang C, Su B, Lu S, Han S, Jiang H, Li Z, Liu Y, Liu H, Yang Y (2021) Effects of glutathione on growth, intestinal antioxidant capacity, histology, gene expression, and microbiota of juvenile triploid *Oncorhynchus mykiss*. Front Physiol 12:784852. 10.3389/fphys.2021.784852 34925074 10.3389/fphys.2021.784852 PMC8680104

[CR135] Yancheva VS, Velcheva IG, Georgieva ES, Stoyanova SG (2019) Periodic acid - Schiff (PAS) reaction in fish liver exposed to fungicide contamination: a possible histochemical biomarker. Ecol Balkan 11(1):1–10

[CR136] Yang J, Zhou WW, Shi DD, Pan FF, Sun WW, Yang PL, Li XM (2023) The interaction between oxidative stress biomarkers and gut microbiota in the antioxidant effects of extracts from *Sonchus brachyotus* Dc. in oxazolone-induced intestinal oxidative stress in adult zebrafish. Antioxidants 12(1):192. 10.3390/antiox12010192 36671053 10.3390/antiox12010192 PMC9854779

[CR137] Younas W, Khan FU, Zaman M, Lin D, Zuberi A, Wang Y (2022) Toxicity of synthesized silver nanoparticles in a widespread fish: a comparison between green and chemical. Sci Total Environ 845:157366. 10.1016/j.scitotenv.2022.157366 35843321 10.1016/j.scitotenv.2022.157366

[CR138] Yousef A, Abu-Elghait M, Barghoth MG, Elazzazy A, Desouky S (2022) Fighting multidrug-resistant *Enterococcus faecalis* via interfering with virulence factors using green synthesized nanoparticles. Microb Pathog 173:105842. 10.1016/j.micpath.2022.105842 36280163 10.1016/j.micpath.2022.105842

[CR139] Zahran E, Mahgoub H, Abdelhamid F, Sadeyen J, Risha E (2019) Experimental pathogenesis and host immune responses of *Enterococcus faecalis* infection in Nile tilapia (*Oreochromis niloticus*). Aquaculture 512:734319. 10.1016/j.aquaculture.2019.734319

[CR140] Zou J, Secombes CJ (2016) The function of fish cytokines. Biology 5(2):23. 10.3390/biology5020023 27231948 10.3390/biology5020023 PMC4929537

