# Supplementary material for: Thyme-synthesized silver nanoparticles mitigate immunosuppression, oxidative damage, and histopathological alterations induced by multidrug-resistant Enterococcus faecalis in Oreochromis niloticus: in vitro and in vivo assays
Source: Fish Physiol Biochem. 2025 Aug 15;51(4):146. doi: 10.1007/s10695-025-01560-5 (PMC12356727; doi:10.1007/s10695-025-01560-5)
Supplement: Supplementary file 1 — Supplementary file1 (DOCX 5353 KB) [file 10695_2025_1560_MOESM1_ESM.docx]

**Supplementary data**

Thyme-synthesized silver nanoparticles mitigate immunosuppression, oxidative damage, and histopathological alterations induced by multidrug-resistant *Enterococcus faecalis* in *Oreochromis niloticus*: in vitro and in vivo assays

**Authors**

**Dalia A. Abdel-moneam ^a^, Hanan S. Khalefa ^b^*, Maha M. Rashad ^c^, Ghada E. Ali ^c^, Yasmine H. Ahmed ^d^ ,** **Eman R. Mohammed** **^e^ ,** **Osama A. Fouad ^f^ ,** **Ramadan A. Geioushy ^f^*, Sahr B. Mahmoud ^g^**

^a^ Department of Aquatic Animal Medicine and management, Faculty of Veterinary Medicine, Cairo University, Giza, 12211, Egypt

^b^ Department of Veterinary Hygiene and Management Faculty of Veterinary Medicine, Cairo University, Giza, 12211, Egypt

^c^ Department of Biochemistry and Molecular Biology, Faculty of Veterinary Medicine, Cairo University, Giza 12211, Egypt

^d^ Department of Cytology and Histology, Faculty of Veterinary Medicine, Cairo University, Giza, 12211, Egypt

^e^ Department of Microbiology, Faculty of Veterinary Medicine, Cairo University, Giza, 12211, Egypt

^f^ Nanostructured Materials and Nanotechnology Department, Advanced Materials Institute, Central Metallurgical Research and Development Institute, P.O. Box 87, Helwan, Cairo 11421, Egypt

^g^ Department of Hydrobiology, Veterinary Research Institute, National Research Centre, Dokki, Giza, Egypt.

***Corresponding authors**

1-Hanan S. Khalefa

Department of Veterinary Hygiene and Management, Faculty of Veterinary Medicine, Cairo University, Giza, 12211, Egypt, [hanansaad04@gmail.com](mailto:hanansaad04@gmail.com); [hanan_saad@cu.edu.eg](mailto:hanan_saad@cu.edu.eg).

2- Ramadan A. Geioushy

Nanostructured Materials and Nanotechnology Department, Advanced Materials Institute, Central Metallurgical Research and Development Institute, P.O. Box 87, Helwan, Cairo 11421, Egypt, [r_gushy@yahoo.com](mailto:r_gushy@yahoo.com)

**Authors’ emails**

[**dr.daliaashraf@gmail.com**](mailto:dr.daliaashraf@gmail.com)

[Maha.Mansour@vet.cu.edu.eg](mailto:Maha.Mansour@vet.cu.edu.eg)

[dr.ghadaalimg@gmail.com](mailto:dr.ghadaalimg@gmail.com)

[yasmine_hamdi@cu.edu.eg](mailto:yasmine_hamdi@cu.edu.eg)

[eman_ragab2008@cu.edu.eg](mailto:eman_ragab2008@cu.edu.eg)

[oafouad@yahoo.com](mailto:oafouad@yahoo.com)

[Dr.sahrbadrawy@yahoo.com](mailto:Dr.sahrbadrawy@yahoo.com)

**Supplementary data Tables: 1**

**Supplementary data figures: 3**

**Table S1** Biochemical characterization of *E. faecalis* strain

| **Biochemical test** | **Results** * |
| --- | --- |
| **Gram staining** | Gram positive cocci |
| **Bile-Esculin agar (black)** | + ve |
| **Voges–Proskauer’s** | + ve |
| **PYR** | + ve |
| **Esculin hydrolysis** | + ve |
| **Arginine utilization** | ND |
| **Glucose** | + ve |
| **Arabinose** | - ve |
| **Lactose** | + ve |
| **Sucrose** | + ve |
| **Sorbitol** | + ve |
| **Mannitol** | + ve |
| **Salt tolerance (6.5 % NaCl)** | + ve |
| **Raffinose** | - ve |
| **ONPG** | - ve |

* +ve: positive result, -ve: negative result; ND: not detected; PYR: L-Pyrrolidonyl-β-naphthylamide test; ONPG: O-Nitrophenyl-β-D-Galactopyranoside Test.


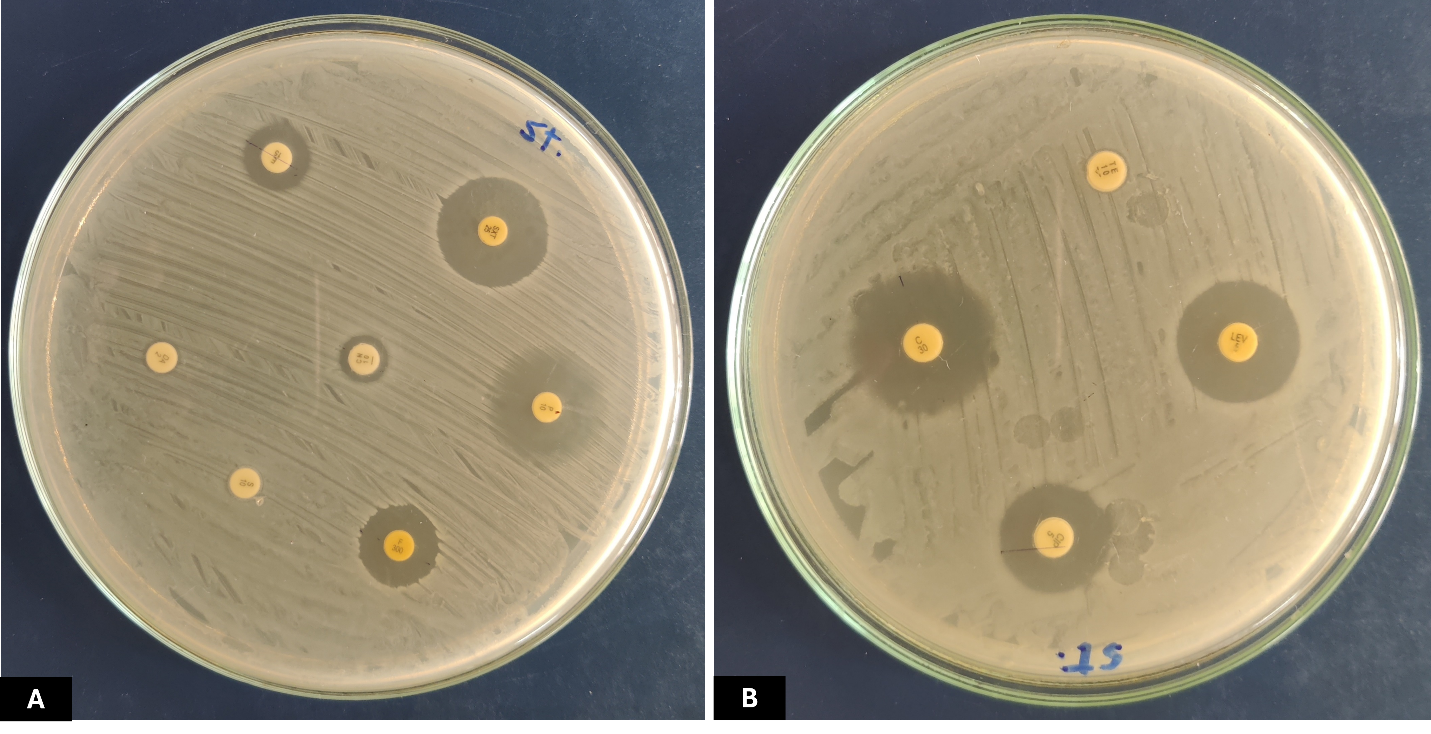


**Fig. S1** The antibiotic susceptibility profile of isolated MDR *E.faecalis*


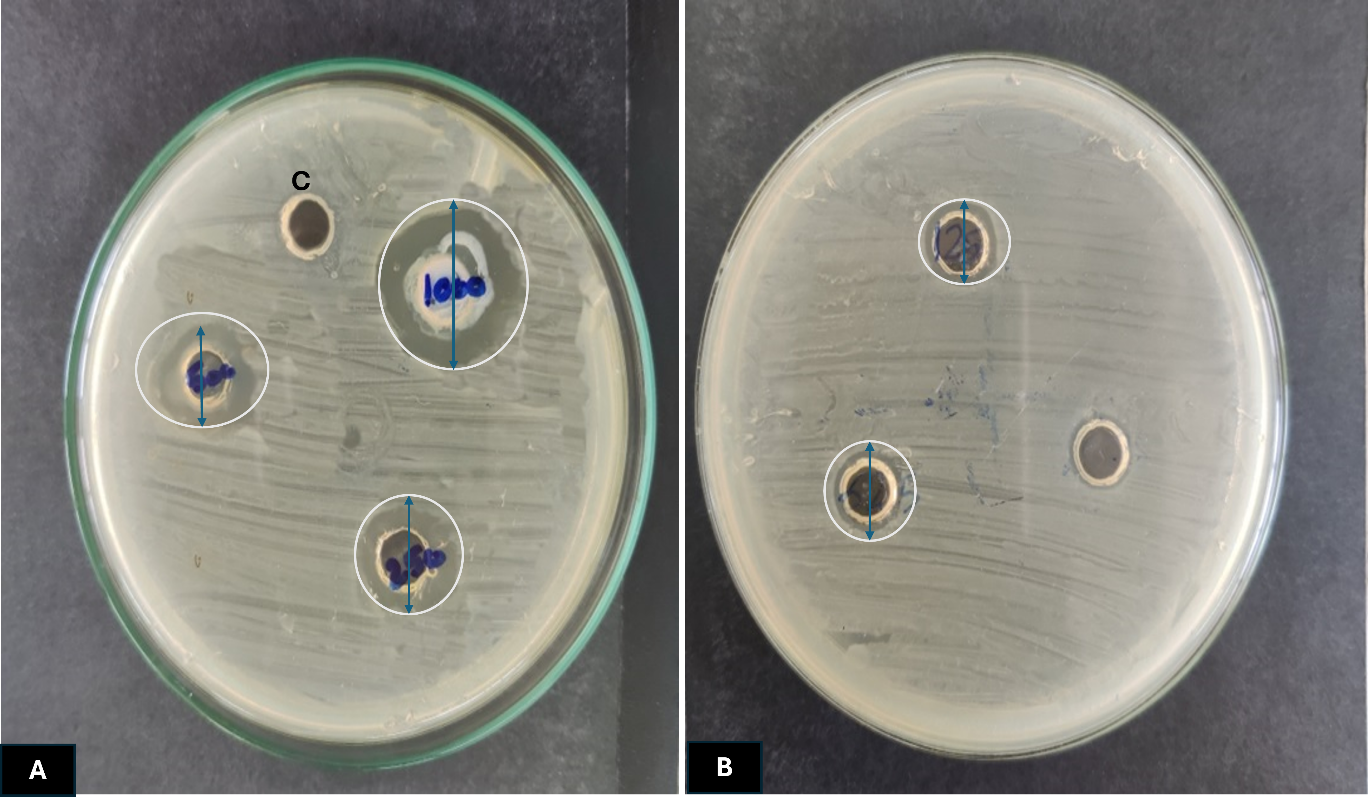


**Fig. S2** The antibacterial potential of thyme-AgNPs against MDR *E. faecalis,* (**A**): Zone of inhibition of the concentrations 1000, 500, and 250 μg/mL, and C was negative control (distilled water). (**B**): Zone of inhibition of the concentrations 125,62.5,and31 μg/mL.


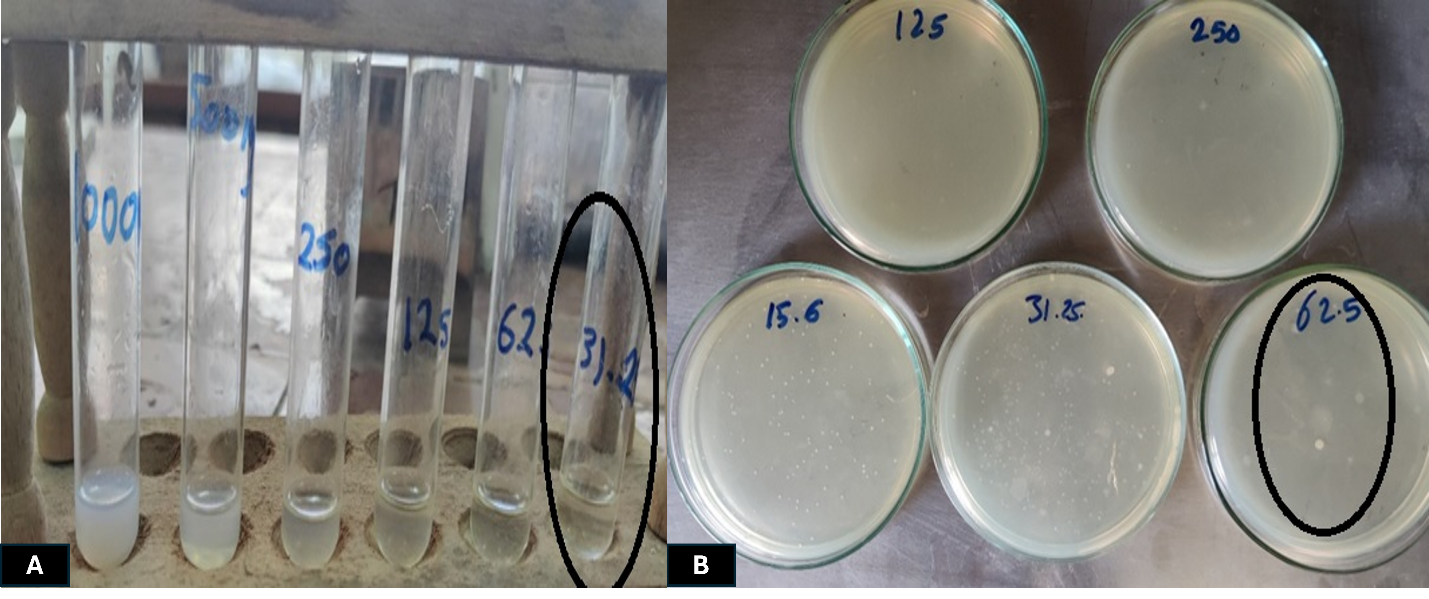


**Fig. S3 (A)** Minimum inhibitory concentration (MIC) of thyme-AgNPs at conc. 31.25 μg/mL, and **(B)** Minimum bactericidal concentration (MBC) of thyme-AgNP at conc. 62.5 μg/mL, against MDR *E.faecalis*
